# Perceptions of caring behaviours among patients, nurses, nursing students: mixed methods systematic review

**DOI:** 10.1186/s12912-026-04600-4

**Published:** 2026-05-09

**Authors:** Nafisa Bardaie, Janet Holt, Gretl A. McHugh

**Affiliations:** 1 School of Healthcare, University of Leeds, Leeds, UK; 2 Healthcare Ethics in School of Healthcare, University of Leeds, Leeds, UK; 3 Applied Health Research in School of Healthcare, University of Leeds, Leeds, UK; 4 Shifa College of Nursing, Shifa Tameer-e-Millat University, Islamabad, Pakistan

**Keywords:** Adult patients, Caring behavior, Compassionate care, Empathy, Experience, Hospitalized patients, Mixed methods, Nurse, Nursing student, Nurse-patient relations perceptions, Quantitative research, Qualitative research, Staff nurse, Student, Systematic review

## Abstract

**Background:**

The foundation and core value of the nursing profession is caring. Worldwide, it is expected that nursing education will cultivate caring behavior amongst nurses to fulfill the health needs of a patient. Three earlier reviews related to caring behavior were identified. However, these reviews were conducted over ten years ago and other literature has since been published. Therefore, it is timely to synthesise the most recent evidence related to the perceptions of patients, nurses, and nursing students of caring behaviours exhibited by nurses.

**Methods:**

A mixed methods systematic review was conducted in accordance with Joanna Briggs Institute (JBI) guidelines. MEDLINE, Web of Science, Scopus, and Cumulative Index of Nursing in Allied Health Literature (CINAHL), PsycINFO, and Embase were searched to identify original peer reviewed studies published from January 2009 onwards. All records were systematically screened for eligibility based on their title, abstract, and full text by all three authors. Eligible studies were critically appraised for methodological quality using the Mixed Methods Appraisal Tool. Both qualitative and quantitative studies were reviewed and data separately extracted for each type of study. The findings were narratively synthesised using a convergent integrated approach. The thematic synthesis of qualitative data and narrative summary of quantitative data was integrated to provide the overall findings of the review.

**Results:**

From 4130 records identified, 44 studies were included (19 qualitative, 23 quantitative, and two mixed methods study). The studies covered several hospital specialty areas. Two themes, physical care with sub-themes of knowledge and skills; comfort; and assurance, and expressive care with sub-themes of connectedness; being respectful; trusting relationships, and teaching and learning emerged from the analysis.

**Conclusions:**

In this review, deficits in knowledge and training requirements of both nurses and student nurses have been identified. For nurse leaders, the review findings will bring insight to those who develop nursing policies to ensure that caring behaviours are incorporated into guidelines and job descriptions for registered nurses.

**Clinical trial number:**

Not applicable.

**Supplementary Information:**

The online version contains supplementary material available at 10.1186/s12912-026-04600-4.

## What is already known

Previous studies have identified the qualities of a good nurses and important caring behaviours of nurses

**Supplementary Information:**

The online version contains supplementary material available at 10.1186/s12912-026-04600-4.

## What this paper adds

This review included more papers with a qualitative design than the previous reviews, and it is possible that more in depth studies of the phenomena of caring revealed more abstract concepts than quantitative papers measuring specific traits were able to achieve.

This review highlights the important concepts underpinning caring behaviour.

The evidence from this review suggests that physical and expressive aspects of care should be incorporated in the nursing curriculum to help nurse educators work with nursing students in cultivating caring behaviours.

**Supplementary Information:**

The online version contains supplementary material available at 10.1186/s12912-026-04600-4.

## Background

The foundation and core value of the nursing profession is caring. Worldwide, it is expected that nursing education will cultivate caring behavior amongst nurses to fulfill the health needs of a patient [1]. Caring behavior has long been recognised as the philosophical and professional foundation of nursing practice. The earliest and most influential scholarly work on caring behavior is attributed to Jean Watson, whose Human Caring Theory positioned caring as the moral and ethical core of nursing rather than a mere technical activity [2, 3]. Watson conceptualized caring as a humanistic, relational process that occurs through meaningful nurse–patient interactions and emphasised the importance of empathy, compassion, presence, and respect for human dignity.

There are two aspects to caring, physical and expressive care. Physical care includes technical, therapeutic and nursing interventions such as medication administration, providing hygiene measures and a calm and comfortable environment. Expressive (also known as psychological) care focuses on compassionate care. Examples include listening to patients, maintaining privacy, showing sensitivity and trustworthiness, and health education for patients and their carers [2, 4, 5]. Similarly, Kristen Swanson’s middle-range theory of caring integrated both dimensions by linking practical nursing actions (doing for) with emotional and relational engagement (being with and maintaining belief). Together, these seminal contributions established that high-quality nursing care requires a balance between physical interventions and expressive interactions [6, 7].

The integration of expressive care in nursing education is important. Nursing institutions place a great emphasis on the cognitive and psychomotor aspects of caring. Although these domains are important for clinical practice, student nurses also need to develop their emotions and feelings towards the patients and strengthen their expressive care [8].

There is evidence in the literature that caring leads to positive quality outcomes for patients and that the nursing curriculum should encompass caring competencies, emotional and expressive aspect of care, alongside professional knowledge and skills [9]. Furthermore, student nurses enrolled in the nursing profession have an optimistic caring vision. This should be reinforced consistently within nursing education [10, 11].

Around the world, nursing education is expected to develop caring practices in the nurses to fulfil health needs of their patients [12]. It is considered a pivotal element of quality healthcare. Furthermore, within the fast-growing system of health care, nurses are challenged by task-oriented approaches, where they may not observe caring behaviours during their encounter with patients. To overcome these challenges, nurses should learn the required knowledge and skills, and build a caring attitude towards their patients.

When patients receive good care, it can have a positive effect and bring a sense of satisfaction into the person’s life [13]. Patients who experience a caring attitude from nurses are found to have a higher level of satisfaction, able to manage their stress, feel comfortable, and confident in their own care. Nurses also experience satisfaction in their personal and professional lives [11, 14].

Three earlier reviews related to caring behaviour were identified [15–17]. The quantitative systematic review [15] compared the perceptions of caring behaviours among nurses and patients while that of mixed methods systematic review [18] identified the qualities of a good nurse. A narrative review identified the most important caring behaviours of nurses [16]. However, these reviews were conducted over ten years ago and other literature has since been published.

Although caring behavior is a core component of nursing practice, existing evidence is fragmented and largely examines single perspectives or isolated methodologies. There is limited integrated synthesis comparing perceptions of hospitalized adult patients, nurses, and nursing students. A mixed-methods systematic review is therefore necessary to consolidate quantitative outcomes and qualitative insights, clarify inconsistencies in perceptions, and generate comprehensive evidence to inform nursing practice, education, and future research.

## Methods

### Design and search strategy

**Fig. 1:**
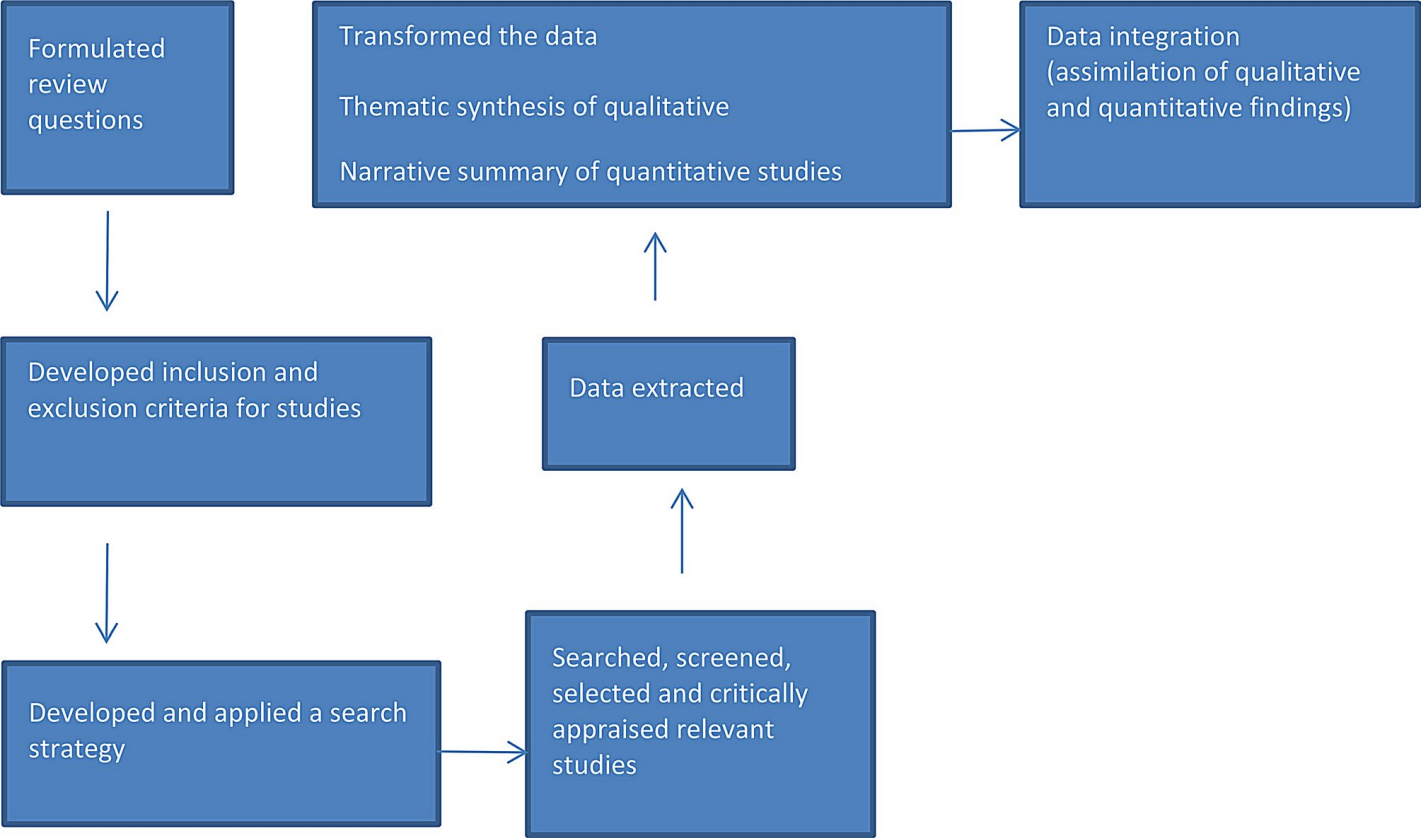
Mixed methods systematic review process

The review is reported according to the Preferred Reporting Items for Systematic Reviews and Meta Analyses PRISMA guidelines [19]. As there were qualitative and quantitative studies exploring the perceptions of caring behavior of nurses, a mixed methods systematic review (MMSR) was undertaken. This review followed the Joanna Briggs mixed methods systematic review approach [20]. A mixed methods convergent integrated approach was considered appropriate for this review, including both qualitative and quantitative research studies, and enabling the transformation and synthesis of both types of studies [21]. Figure 1 provides an overview of the review process. The following electronic databases were initially searched for relevant papers in September 2019 and then again in December 2025: Medline (Ovid), Embase (Ovid), PsycINFO (Ovid), Web of Science, Scopus, and Cumulative Index of Nursing in Allied Health Literature (CINAHL). The inclusion criteria and keywords were formulated using the SPIDER approach, that is, defining, Sample, Phenomenon of interest, Design, Evaluation, and Research type [22]. The searches were conducted by one reviewer using key terms and MESH headings related to ‘adult patient’, ‘caring’, ‘caring behavior’, ‘compassionate care’, ‘empathy’, ‘experience’, ‘hospitalized patients’, ‘mixed methods’, ‘nurse’, ‘nurse patient relations’, ‘perceptions’, ‘quantitative research’, ‘qualitative research’, ‘staff nurse’, ‘student’ (see Additional file 1 for full search strategy). Additionally, searches were limited to studies published in English. Hand searching of reference lists of identified papers was also undertaken to check for the additional papers.

#### Eligibility criteria

For this mixed methods review, quantitative, qualitative, and mixed methods studies were included. The diverse designs have the potential to offer valuable insights into individuals’ experiences and perspectives [23]. This review included studies published from January 2009 onwards. The significant conceptual and measurement developments in caring behaviors occurred after 2008, including refinement of caring constructs and wider use of standardized instruments across diverse cultural settings. Limiting the search to 2009 onward allowed us to capture contemporary understandings of caring behaviors that reflect current nursing education, patient expectations, and healthcare delivery systems. Secondly, the healthcare environment has undergone considerable transformation over the past decade, including increased emphasis on patient-centered care, technological integration, and professional role expansion. Studies published prior to 2009 were conducted within substantially different healthcare contexts, which may limit comparability with more recent findings. Lastly, the previous review covered literature up to 2008 [15]; therefore, our timeframe was intentionally selected to avoid duplication while providing an updated synthesis of evidence. Our review thus functions as an extension and update rather than a replication. Studies that met the eligibility criteria outlined in Table 1 were included.

**Table 1:** Studies included in review

Author(s) & country	Setting or context	Aim(s) of the study	Design & sample
Afaya et al., [14] Ghana	Medical-Surgical ward	Assess patients perception of caring behavior	Descriptive cross-sectional study Patients (*n* = 183)
Akansel et al., [24] Turkey	One university hospital	Assess nurses’ perception of caring activities	Cross-sectional descriptive method Nurses (*n* = 260)
Aktas and Karabulut, [25] Turkey	Nursing school	Identify correlation of the undergraduate student nurses ‘professional values and their caring behaviour	Cross-sectional descriptive survey design (*n* = 351) undergraduate nursing students (years 1 and 4)
Allari et al., [26] Middle East	Nursing school	Compare the perception of undergraduate student nurses about caring.	Cross-sectional-descriptive, comparative design. Nursing students (*n* = 1,582)
Ambrosi et al., [27] Italy	Nursing school	Explore perception regarding caring behaviour among student nurses.	Qualitative using the longitudinal approach. Student nurses (Years 2 and 3) (*n* = 24)
Andersson et al., [28] Sweden	Coronary Care Unit	Registered nurses’ perception of the caring concept.	Qualitative using Phenomenology approach. Nurses (*n* = 16)
Aupia et al., [29] Indonesia	One Hospital and one nursing school	Comparison of the perception of caring behavior among nurses, patients and student nurses.	Descriptive comparative study (*n* = 159) (53) nurses, (53) patients, and (53) student
Canzan et al., [30] Italy	Hospital setting (gerontological department	Comparison of the perception of caring from nurses and patients.	Qualitative using descriptive approach /(*n* = 40) (20) nurses and (20) patients
Cheruiyot and Brysiewicz, [31] South Africa	Inpatient (Rehabilitation setting)	Explore perception of caring and uncaring nursing encounters.	Qualitative using descriptive approach. Nurses (*n* = 21)
Costello, [32] Boston	Medical Surgical unit	Identify the characteristics and behaviour of nurses who were identified by the patient as the best nurses.	Qualitative using descriptive approach. Nurses (*n* = 9)
Coughlin, [33] United States (Northeast)	Hospital setting	Explore perception of nurses and patients about care during key events of the hospitalisation (admission, transfer to the operation room and preparation for discharge.	Qualitative using ethnographic approach /(*n* = 12) (10) patients and (2) nurses
Dobrowolska and Palese, [10] Eastern Region of Poland	Nursing Institution	Student nurses perception of caring, its features and possible hurdles.	Qualitative using descriptive approach. Polish students of three years degree programme (*n* = 15)
Edvardsson et al., [34] Australia	Tertiary acute care setting	Association of patients’ perspectives about the caring behaviour and person- centredness with the outcome of quality care nursing.	Descriptive non-experimental correlational design /Patients (*n* = 210)
Esmaeili et al., [31] /Tehran	Cardiac Unit	Explore cardiac patients’ perception of patient-centred care.	Qualitative using descriptive approach. Cardiac patients /(*n* = 18)
Fang et al., [35] East Coast of China	Internal medicine department, Endocrinology, Respiratory, Cardiology, Gastroenterology, Pain, Neurology, Nephrology, Oncology, and Hematology	Perception of caring based on the ‘CARE model’ from nurses. To provide practical guidelines for nurses to improve their behaviour.	Cross-sectional descriptive method Nurses (*n* = 157)
Fenizia et al., [36] Italy	Nursing school	Explore variations in caring behavior among student nurses during the academic year.	Descriptive longitudinal Undergraduate student nurse (years 2 and 3) (*n* = 103)
Ferri et al., [37] Italy	Nursing school	Assess perception of caring behavior by student nurses.	Three-cohort observational study. Nursing students (*n* = 331)
Ferede et al., [38] Ethiopia	Multicenter Medical-surgical	Explore the factors influencing nurses’ perceptions of caring behaviors and to develop a deeper and more holistic understanding of these perceptions.	Sequential explanatory mixed-methods. Nurses. Quantitative (*n* = 148) qualitative (*n* = 12)
Flynn, [39] UK	Acute hospital trauma ward of the orthopaedic department	Assess perception of caring from both patients and healthcare professionals	Descriptive study (*n* = 83) Patients (30) and (53) Healthcare Professionals (doctors, nurses, physiotherapists and occupational therapists
He et al., [40] China (Central, Southern, and Eastern)	Five hospitals in southern, central and eastern China Each three medical surgical unit	Comparison of the perspectives among nurses and patients about caring behaviour.	Descriptive comparative survey. Patients and nurses (*n* = 1220)
Papastavrou et al., [41] Six different European countries (Finland, Greece Cyprus, Czech Republic Hungary, Italy)	Surgical Unit	Assess perception of patients and nurses about caring behaviour.	Descriptive comparative survey (*n* = 2854) (1659) surgical patients and (1195) nurses
Pearcey, [42] United Kingdom	Hospital setting	Explore opinion of qualified nurses about the central values in clinical nursing.	Qualitative using grounded theory approach. Qualified nurses (*n* = 25)
Petrou et al., [43] Cyprus-Italy	Nursing Department of Cypriot University	Explore student nurses perception of caring.	Qualitative using descriptive approach. Students (*n* = 122)
Phillips et al., [44] United Kingdom	Nursing Institution	Student nurses’ beliefs and values about caring at the initiation of the nursing programme.	Qualitative using the longitudinal approach. Undergraduate pre-registration nursing students from the two discrete programmes (Advanced Diploma and BSc (Honours) (*n* = 36)
Jardien-Baboo et al., [45] South Africa	Public hospital	Explore perception about patient-centred care from the professional. Enabling and inhibiting factors to patient- centered care.	Qualitative using descriptive approach. Nurses (*n* = 40)
Kalfoss and Owe, /Oslo	Cancer Nursing, Nephrology Nursing, Pastoral Counselling, Public Health Nursing, and Masters’ students in Community Health Nursing	Explore concept of professional care from student nurses.	Qualitative using exploratory approach. Post-bachelor students (*n* = 31)
Kiliç and Öztunç, [46] Turkey	Surgical operation department	Comparison of the perception of patients and nurses.	Descriptive study (*n* = 449) (379) Patients and (70) nurses
Labrague, [47] Catbalogan City, Philippines	Different clinical units of Samar Provincial Hospital	Assess perception of patients towards caring competencies of Level IV students.	Cross-sectional descriptive method (*n* = 174 patients)
Labrague et al., [11] Philippines Greece, Nigeria, India	Nursing school	Describe caring behavior of student nurses in the four countries.	Descriptive comparative survey design. Nursing students (*n* = 467)
Li et al., [12] Taiwan	Nursing school & Clinical setting (medical, surgical, obstetric, paediatric and intensive care units)	Comparison of the views about caring behavior among students and registered nurses.	Cross-sectional study (*n* = 647) Participants from the three programmes (330) nursing students: Five-year ADN programme, the two-year and four-year baccalaureate degree of nursing programmes. (317) registered nurses
Mako et al., [48] Sweden (South)	Surgical department	Patients’ meaning of good care.	Qualitative using grounded theory approach patients (*n* = 13)
Marshall et al., [49] South Australia	Metropolitan Hospital (surgical unit)	Understanding of patients about patient-centred care and identifying its relationship it with the existing literature.	Qualitative using phenomenology approach Patients (*n* = 10)
Merrill et al., [50] Ghana	Medical- surgical ward(Trauma centre)	Describe patients’ perceptions of caring behaviour.	Descriptive cross-sectional study. Patients (*n* = 105)
Mlinar, [9] Slovenian Rou	Nursing school	Identification of the significant differences in the mean scores among the first-year and third-year student nurses.	Cross-sectional descriptive method. First-year and third-year nursing students (*n* = 166)
Modic et al., [51] Midwest	Acute care setting	Diabetic patients and nurses perception of caring behaviour.	Qualitative using descriptive approach (*n* = 118) (64) Nurses and (54) patients with diabetes
Omari et al., [52] /Jordan	Coronary care unit	Describe perception of patients and nurses about caring behaviour. Comparison of perceptions among both groups	Descriptive comparative design (*n* = 210) (150) Patients and (60) nurses
Rahman et al., [53] Pakistan 2019	Tertiary care hospital (Orthopaedic Department	Health care providers and patients. Perception regarding best practices in patient-centred care (PCC). Identified the similarities of perspectives between both groups.	Qualitative using descriptive approach (*n* = 36) (18) Healthcare providers (nurses, consultant doctors, Residents, radiologists, and physiotherapists) and (18) patients
Roulin et al., [54] Geneva, Switzerland	Rehabilitation	Describes and compares nurses’ and admitted patients’ perception of caring behaviors	Comparative descriptive design Nurses (*n* = 34) Elderly (*n* = 64)
Sundus and Younas, [55] Pakistan	Medical Surgical Departments of three private hospitals in Islamabad, Pakistan	Patients’ perspective of caring behaviour of male nurses.	Descriptive qualitative study drawn from a larger convergent mixed methods approach. Patients (*n* = 15)
Thomas et al., [56] North Texas	Long-term acute care hospital	Congruency of perceptions of nurse caring behaviour between patient and nurse. Determine patient perception changes over time.	Mixed methods Triangulation Design Convenience Patients (*n* = 25) Nurse= (*n* = 85)
Trinidad et al., [57] Universidad Europea de Madrid	Nursing School	Undergraduate student nurses’ perception of caring and identified any differences in the behaviour among them.	Cross-sectional design. Undergraduate nursing students (*n* = 321)
Tsai and Wang, [58] Taiwan (Southern)	Hospital setting	Describe perception of registered nurses of caring behaviour.	Qualitative using descriptive approach. Nurse (*n* = 58)
Youssef et al., [59] Taif City	Medical-Surgical	Describe perception of caring behavior from nurses.	Quantitative descriptive correlational design. Nurse (*n* = 90)
Zamanzadeh et al., [60] Tabriz and Urmia faculties of nursing	Nursing School	Perception of student nurses toward caring behaviour.	Cross-sectional descriptive method. All first and fourth-year nursing students (*n* = 230)

**Fig. 2:**
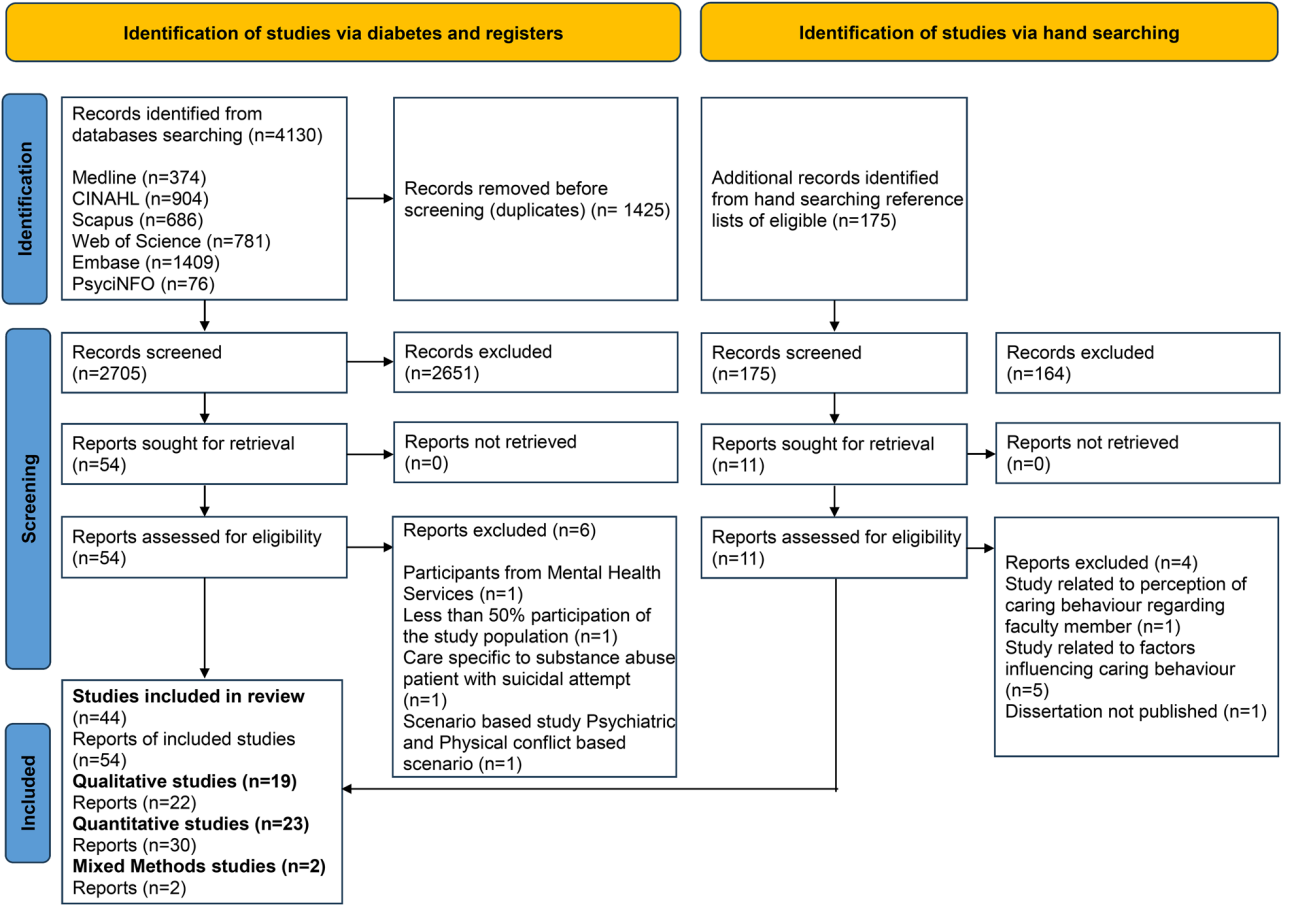
Prisma flow chart

This literature search retrieved 4130 articles. After the removal of duplicates (*n* = 1425), the records were screened by title (*n* = 2730), abstract (*n* = 150) and full text (*n* = 53) by all three authors and discrepancies were resolved to reach a consensus. Consensus was reached for each decision in the screening process and identified potentially relevant studies for the final inclusion in the review. A total of 44 studies were selected for appraisal of the methodological quality. The search strategy and selection process are presented in a PRISMA flow chart in Fig. 2 [19].

### Outcome of the study and operational definition

The outcome of this study was to synthesize how caring behaviours are understood and perceived by hospitalized adult patients, nurses, and nursing students, highlighting common and divergent perceptions across stakeholder groups. Caring behaviours refer to observable and perceived actions, attitudes, and interactions demonstrated by nurses or nursing students that convey empathy, respect, compassion, presence, and professional competence during patient care, as reported in included studies. Perception is defined as the subjective understanding, interpretation, or evaluation of caring behaviours by patients, nurses, or nursing students, captured through qualitative narratives or quantitative measurement tools. Hospitalised adult patients are individuals aged 18 years or older who are admitted to healthcare facilities and receive nursing care, as defined in the reviewed studies. Nurses are licensed nursing professionals involved in direct patient care within hospital settings, regardless of clinical specialty or years of experience. Nursing students are individuals enrolled in undergraduate nursing programs who provide patient care during clinical placements or training. A mixed-method systematic review is a research approach that systematically identifies, appraises, and synthesizes quantitative, qualitative, and mixed-methods studies to develop a comprehensive understanding of perceptions of caring behaviours.

### Data extraction and quality assessment

The lead reviewer extracted data of each study using a proforma. Data included general study information such as the author’s name, year of publication, context, aim, methodology, key findings relevant to the research questions, and limitations of the studies. In addition, two reviewers verified the data item of the included studies. The discrepancies were resolved to reach a consensus among all the reviewers. A total of 44 original studies were screened for the quality assessment by lead reviewer using the Mixed Methods Appraisal Tool (MMAT) [61]. This tool is specifically designed for use across qualitative, quantitative and mixed methods designs. The MMAT comprises of five categories of study designs with methodological quality criteria. Reviewers rated studies based on the relevant criteria, assigning “Yes” if the criterion was met, “No” if not met, or “Can’t tell” if there was insufficient information. Obtaining a “Yes” rating only indicates high methodological quality of the studies. Following MMAT guidance, no overall scores were computed. Two reviewers verified the methodological quality of the included studies. The study summary and methodological quality of the studies are presented in Tables 1 and 2. Regardless of the methodological quality, all the studies were included in the review.

**Table 2:** Methodological quality of the study

Qualitative studies	Yes	No	Can’t tell
Is the qualitative approach appropriate to answer the research question?	19		
Are the qualitative data collection methods adequate to address the research question?	19		
Are the findings adequately derived from the data?	19		
Is the interpretation of results sufficiently substantiated by data?	19		
Is there coherence between qualitative data sources, collection, analysis and interpretations?	19		

Quantitative studies	Yes	No	Can’t tell
Is the sampling strategy relevant to address the research question?	21	1	1
Is the sample representative of the target population?	22	0	1
Are the measurements appropriate?	14	5	4
Is the risk of nonresponse bias low?	10	13	0
Is the statistical analysis appropriate to answer the research question?	23	0	0

Mixed methods study	Yes	No	Can’t tell
Is there an adequate rationale for using a mixed methods design to address the research question?	1	1	
Are the different components of the study effectively integrated to answer the research question?	2		
Are the outputs of the integration of qualitative and quantitative components adequately interpreted?	2		
Are divergences and inconsistencies between quantitative and qualitative results adequately addressed?	2		
Do the different components of the study adhere to the quality criteria of each tradition of the methods involved?	1		1

## Data analysis

A convergent integrated approach was used to synthesise and integrate the data [20]. For the qualitative studies, a thematic analysis was conducted. This approach is recognised for use in secondary data synthesis of primary qualitative studies [27]. Three phases of analysis were undertaken: firstly, codes were identified ‘line by line’ from the findings and discussion sections, the development of ‘descriptive themes’, and the generation of ‘analytical themes’ [27]. In order to keep descriptive themes aligned with those of the primary studies, the researcher used the subscales of caring behaviour instruments. For the descriptive themes, the subscales identified were, ‘knowledge and skills’, ‘assurance’, ‘connectedness’, ‘being respectful’ from the ‘Caring Behaviour Instrument’ (CBI − 24) [62], ‘comfort and trusting relationships’ from ‘Care Q-50’ [63], and ‘teaching and learning’ from ‘Caring Behaviour Assessment’ (CBA-63) [64]. The codes were organised into descriptive themes under two analytical themes: physical care and expressive care. For the synthesis of quantitative data, data were extracted and converted into textual descriptions or narrative interpretations to allow integration with the qualitative findings [20, 65]. The thematic synthesis of both qualitative and quantitative data was integrated to gain a comprehensive understanding of the findings, and these were grouped under two broad themes of physical and expressive care.

## Results

### Search result and study characteristics

A total of 4130 non-duplicate records were identified from the database searches. A further 110 records were identified from hand searching. Overall, forty-three studies met the eligibility criteria, and were included in the review (Fig. 2). Out of these, 19 were qualitative studies, 23 were quantitative studies and two mixed methods study.

These studies were conducted in different countries: 18 from European countries, 14 from Asia, four from America, four from Africa, two from Australia, and one involving multiple countries (Philippines, Greece, Nigeria, and India). All studies were published in English. Most of the studies were conducted in medical and surgical departments with others involving gerontology, acute care, cardiac care, and rehabilitation departments.

The study designs included: qualitative descriptive, grounded theory, phenomenology, and longitudinal studies and investigated nurses’ perceptions about caring (*n* = 14), and descriptive and phenomenology studies explored the perceptions regarding patient-centred care (PCC) (*n* = 5). One ethnographic study explored the perceptions of nurses and patients about care during key events of hospitalisation (admission, transfer to the operation room and preparation for discharge). The quantitative cross-sectional descriptive studies investigated nurses’ perceptions of caring behaviour (*n* = 13), the descriptive longitudinal study analysed the variations in caring behaviour among student nurses during the academic year (*n* = 1), the cross-sectional comparative study and the mixed methods triangulation designed studies explored the perceptions among nurses, nursing students and patients (*n* = 8), and the correlational study explored the association of patients’ perspectives about caring behaviour and person‐centeredness with the outcomes of quality care nursing (*n* = 1).

The Mixed Methods Appraisal Tool (MMAT) [61] was used for the methodological appraisal of the studies. There were a number of methodological strengths shown in the individual studies. For example, one qualitative study by Jardien-Baboo et al., triangulated the data by use of semi-structured interviews, focus groups, observations and field notes [66]. Triangulation provides various perspectives on the same phenomenon and increases the credibility of the findings [45]. Another study conducted in the Urdu language used back-to-back translation of the data [67] and a further study piloted the interview guide before use and achieved data saturation [53].

Some quantitative studies mentioned the reliability and validity of the instrument used in previous studies [9, 42, 54]. Some studies calculated reliability and validity [11–13, 26, 29, 35–37, 39–41, 50, 52, 57]. Some studies mentioned the response rate [9, 11, 12, 14, 26, 29, 36, 37, 42, 57]. Only one quantitative study used the Caring Behaviour Assessment (CBA) instrument which was translated into Arabic and the researchers applied back-to-back translation [41]. In another study, a Caring Behaviour Scale (CBS) was developed to collect data relevant to the Taiwanese culture [12].

There were a number of methodological limitations noted in the studies. Several qualitative studies did not describe the methods used which helps to ensure the trustworthiness of the data [30, 32, 44, 49, 51, 53, 59]. One study did not describe pilot testing of the interview guide [58].

The majority of the quantitative studies did not describe any pilot testing of the instruments [9, 14, 25, 34, 40, 47, 58], reliability and validity of the instrument [14, 25, 34, 47, 58, 60] and the response rate [13, 25, 34, 35, 39–41, 47, 50, 54, 56, 58, 60]. In one study, the sample size was not identified [68].

Most of the quantitative studies [9, 13, 14, 36, 37, 39, 40, 56, 58, 69] collected data using the Caring Behaviours Inventory (CBI-24) [62]. Some studies used other instruments with similar or different subscales.

In eleven of the qualitative studies, semi-structured interviews were conducted [28, 31, 32, 44, 48, 51, 53, 59, 66, 70, 71]. Researchers [55] used both semi-structured interviews and observations. Three studies collected data using focus groups [30, 46, 67]. The remaining studies used different data collection methods. Data was collected through a survey with open-ended questions [43, 49]; text diaries [10]; participant observations; unstructured interviews [33], and brief stories of patients and family encounters [60].

The synthesised findings are described under two broad analytical themes, physical care and expressive care. Both contain further descriptive themes derived from the literature as explained in the data analysis section above.

## Theme 1: Physical care

This overall theme included three descriptive themes: knowledge and skills, comfort, and assurance.

### Knowledge and skills

In several quantitative studies, participants reported the highest score in the subscale of knowledge and skills as compared to the other subscales [14, 25, 26, 37, 39, 40, 47, 50, 52].

The researchers reported different caring behaviours under this category. For instance, nurses should have up to date knowledge about patients’ health conditions [29, 46, 70]. This enables nurses to make rational decisions about patient care [36, 46, 51]. Providing patients with up to date information enables them to make informed decisions [70]. Moreover, nurses need to protect patients from harm [43]. Protecting patients from harm reflects nurses’ ethical and professional responsibility to ensure safety and prevent adverse outcomes. Nurses need to address patient individual needs [10, 33, 38, 40, 51, 53, 55]. Addressing individual patient needs demonstrates responsiveness and reinforces person-centred care by aligning nursing actions with patient priorities. Nurses need to be competent and confident in their nursing skills [40, 51]. Clinical competence and professional confidence are central to safe care delivery and strengthen patients’ trust in nurses’ ability to manage their health effectively. In this category, nurses went beyond their job descriptions [32] and provided invisible care, for example, planning, reflecting, and critical thinking [48, 59].

Overall, findings suggest that nurses place greater emphasis on knowledge-based and technical competencies than on expressive or relational aspects of care. This reflects a perception that clinical expertise, patient safety, informed decision-making, and skill competence are central to effective nursing practice. Such behaviours demonstrate a task-oriented understanding of caring, where nurses often extend their responsibilities to meet individual patient needs through clinically focused and less visible forms of care.

### Comfort

Considering a patient’s physical comfort is another aspect of physical care [29, 32, 38]. The patients, student nurses, and nurses from the coronary care, gerontological, medical and surgical departments described comfort as performing daily routine activities for the patients. For example feeding, hydrating, bathing, toileting, and administering medication [10, 40, 43]; providing oral care [44]; offering things, such as blankets, position changes, back rubs, adjusting the lighting, and keeping things within easy reach. Other study findings identified activities such as making the surroundings neat and clean before leaving the room as caring behaviours [10, 44, 51].

Physical comfort was commonly perceived as a core element of caring, encompassing assistance with routine daily activities and symptom relief. Across patients, nurses, and nursing students, caring behaviours included meeting basic needs, ensuring hygiene, administering treatments, and maintaining a comfortable and safe environment through small yet meaningful actions that enhanced patient comfort and well-being.

### Assurance

Caring behaviours included ensuring patient safety by protecting them from physical and psychological harm such as falls and been anxious. Ensuring protection from physical risks and psychological distress reflects nurses’ responsibility for patient safety and emotional security. Ensuring administration of medication on time [10] and monitoring the outcomes of medication administered [57]. Administering medications on time demonstrates clinical accountability and reduces the risk of preventable complications. Ongoing evaluation of medication effects highlights the nurse’s role in safeguarding treatment effectiveness and patient safety. Nurses respond to patients promptly [32, 38, 71]. Responding promptly to patient needs reinforces trust and signals attentiveness and reliability in care delivery. Nurses need to fulfill their promises with the patients [59]. Honoring commitments strengthens credibility and promotes a sense of dependability in the nurse–patient relationship. Nurses need to assure them that they are not alone [10]. Providing reassurance addresses emotional vulnerability and reduces feelings of isolation during illness.

## Theme 2: Expressive care

This overall theme included four descriptive themes: connectedness, being respectful, trusting relationships, and teaching and learning.

### Connectedness

The concept of connectedness infers that regardless of the power imbalance, patients believe that they have a mutual relationship with nurses [32]. The caring behaviours identified under this theme are: listening to the patient concerns and resolving their queries [31, 43, 60]. This behavior enables understanding of patient needs, reduces anxiety, and supports informed care decisions and familiar with verbal and non-verbal communication [29, 30, 32]. This may help nurses recognize emotions and unspoken needs, strengthening nurse–patient relationships. Nurses use humor in conversation [32, 71]. This may reduce stress of the patient and promotes a relaxed, supportive care environment when used appropriately. Nurse needs to instill hope in patients [28]. Instilling hope may encourage emotional well-being and motivates patients to engage positively in their care. While communicating nurses may provide therapeutic touch to the patients [43, 53]. Therapeutic touch may convey comfort, reassurance, and empathy, addressing patients’ emotional needs. Nurses need to involve in advocacy of patient [29]. This behavior ensures patients’ rights, preferences, and best interests are protected. Nurses need to involve patients in planning their care [32, 48]. This promotes autonomy, shared decision-making and individualized care. Nurses can share their own health experiences. This may build rapport and reassurance when used professionally and selectively. Nurses need to know the patient by being present with them [30]. Being present with the patient may demonstrates attentiveness and helps nurses understand patients as individuals. Effective communication fosters patient confidence, enabling individuals to voice their concerns freely; however, in its absence, patients may withhold even essential information such as pain-related needs [38]. Connectedness reflects a relational aspect of caring in which patients perceive a sense of mutuality with nurses despite inherent power differences. When such communication is strong, patients feel encouraged to express concerns openly; however, weak connectedness may inhibit disclosure of important needs with potential implications for quality and safety of care.

### Being respectful

The findings from the studies found that being respectful means considering the feelings [29, 38], preferences, emotions, and expectations of patients [25, 31, 48]. This acknowledges emotional experiences and promotes emotional comfort and trust. Nurses need to respect patient autonomy (and that of their families) to make independent decisions regarding their health conditions [12, 31, 71]. Nurses should allow patients to actively participate in self-care activities [10, 43, 59]. This may enhance independence, confidence, and engagement in recovery. Nurses need to fulfill the spiritual needs of patients [10, 29, 46] and acknowledge cultural values and beliefs [28, 43, 66] for their inner comfort. Nurses need to show empathy [38, 51, 54, 55], and respect patient privacy [42, 54]. The findings suggest that respect in nursing care extends beyond courteous behavior to encompass recognition of patients’ emotional, cultural, spiritual, and decisional needs. Collectively, these behaviors highlight respect as a multidimensional construct that strengthens trust and promotes patient engagement, with important implications for person-centred and ethically sound nursing practice.

### Trusting relationships

Building a trusting relationship with the patient was seen as important [9, 33]. This concept describes the need for nurses to respect patient confidentiality [11, 36, 57], as well as developing a trusting relationship by paying attention to the patient’s needs and focusing on their best interests [46].

Trust-building was identified as a fundamental caring behavior, grounded in maintaining patient confidentiality and consistently prioritizing patients’ needs and best interests. By demonstrating reliability, attentiveness, and ethical responsibility, nurses fostered trusting relationships that supported effective and compassionate care.

### Teaching and learning

A nurse should provide information to patients and their families about the person’s state of health, treatment, and how to manage their day-to-day health issues [70]. This promotes self-care and allows the patient to make informed decisions about their health [31, 41, 71]. Providing patients and their families with clear information about health status, treatment, and daily care promotes self-care and empowers patients to make informed decisions regarding their health.

## Discussion

This MMSR has synthesised evidence of the perceptions of caring behaviours among patients, nurses, and nursing students providing an in-depth and comprehensive understanding of the caring phenomena.

The methodological quality of the studies varied but was generally better for qualitative studies. Most of the qualitative studies (*n* = 19) explored the caring concept, described patient-centered care, the concept of professional care, the meaning of good care and the characteristics of the nurses. The quantitative studies (*n* = 23) identified the highest and lowest ranking of caring behaviours and compared them among nurses and patients. The mixed methods study explored the congruency of perceptions of nurses’ caring behaviours between patients and nurses. Another mixed methods study explored nurses perception of caring behavior.

The majority of the studies highlighted the importance of physical and expressive care. However, the emotional and expressive aspects of care appeared to be less frequently discussed. Expressive care means a nurse needs to establish effective communication skills, be respectful towards patients’ needs and preferences, build a trusting relationship and provide information to patients and their families about their health conditions and treatment plans.

This review describes a broad range of caring behaviours for effective patient care. It highlights the important concepts underpinning caring behavior which were not explored in the previous reviews. For example, nurses should have meta-skills such as reflecting and use critical thinking to address patient problems and how to seek solutions. This may enable nurses to assess patient need and help them to make rational decisions.

Additionally, this review emphasises the cultural, emotional, and relational aspects of nursing practice. It highlights a patient-centered approach, recognizes that patients are individuals with unique backgrounds, preferences, and values which may assist nurses to shape their care plans to meet specific individual patient needs. This review also emphasises the importance of empathy, compassion and respect in establishing meaningful relationships with patients and their families. Patients who sense that nurses genuinely care about their wellbeing can lead to improved patient satisfaction and ultimately better health outcomes [13]. Additionally, the papers in this review suggest that patient dignity is upheld through involving a patient in planning their self-care activities and the consideration of the spiritual aspect of care, which too may increase patient satisfaction with the care provided. Trust was seen as a foundation of the nurse-patient relationship, built through maintaining confidentiality and transparent communication. In addition, using humour while providing care to the patients and inspiring hope in patient’s life are also identified. In this review we found that there was a perception that nurses need to protect patients from psychological harm, (such as reducing anxiety) while performing invasive procedures [32]. This is in contrast to the previous review by Papastavrou, Efstathiou 15 where nurses did not identify patient anxiety and depression as specific phenomena. A caring nurse knows how to talk to patients in simple terms about their disease, treatment plan, and the side effects of their medications which helps to alleviate fear and anxiety [17].

A strength of this review is that it included different qualitative designs (phenomenology, grounded, ethnography, longitudinal) to explore variations in the conceptualisation of the phenomenon of caring. In contrast, the previous reviews mostly focused on papers with quantitative designs.

However, there were some similarities between this review and the findings from the previous reviews. Participants across a majority of the studies rated the knowledge and skills of nurses the highest. This finding is consistent with the findings of a previous systematic review based of 29 studies, which compared the perceptions of caring behaviours among nurses and patients [15]. It is also in line with the findings from an earlier mixed methods systematic review of 12 studies which identified the quality of a good nurse [17].

The previous reviews also highlight the importance of nurses staying informed and up to date about their patients’ medical conditions, which is also a feature of this review. Such knowledge equips nurses to promptly recognise potential complications, make an informed decisions regarding patient care, meet their needs, and exhibit competence and confidence in delivering care to the patients. Ultimately, this can lead to enhanced patient well-being.

Similar to the findings in the previous reviews, we found that there was a perception that having adequate knowledge and skills may enable nurses to provide physical comfort for patients. Physical comforts include assisting patients in feeding, hydrating, bathing, toileting [39, 40, 43], changing a patient’s position [60], and providing oral care [44]. A previous review including older patients considered that physical care included helping them take a bath, assisting them to the toilet, and offering a bedpan or urinal promptly [17]. Taking into account a patient’s physical well-being is another facet of providing physical care. Compassionate nurses have a vital role in assisting patients with their everyday tasks and creating a comfortable environment [38].

In order to respond promptly to the needs of the patients, nurses in this review encouraged patients to call in case of problems and, that they should respond to calls quickly [36, 39]. These findings corroborate with a previous review [17]. This caring gesture may help to fulfill patient needs immediately and promote comfort for the patients.

While providing physical care for patients, nurses should be expressive in their responses. They can be expressive towards patients by spending quality time and demonstrating effective communication with them [38]. This may allow patients to express their feelings about their condition and life experiences [31, 43, 51, 71]. These findings are consistent with a previous review [17]. Nurses should be mindful of both their verbal and nonverbal interactions with patients. It is important that they use a gentle and friendly tone while maintaining a warm, approachable facial expression. It is crucial to treat patients with dignity, as doing so can foster trust and ease, encouraging them to openly communicate with nurses.

While it is unsurprising that perceptions of caring behavior still have a strong focus on providing physical care, it is interesting that there appears to be a more clearly defined understanding of the importance of expressive care in the papers selected for this review than in previous reviews. The reasons for this are difficult to define, but maybe explained by an explicit understanding of the importance of compassion in nursing as evidenced by the substantial literature on the subject. *Being compassionate is part of the professional identity that nurses develop because compassion is so central to our understanding of what good nursing is* [72] (p.151). This review also included more papers with a qualitative design than the previous reviews, and it is possible that more in depth studies of the phenomena of caring revealed more abstract concepts than quantitative papers measuring specific traits were able to achieve. However, while expressive care was identified as a feature of caring, perceptions of physical care remain the dominant voice.

The findings of this review contribute to a body of knowledge related to caring behaviours in the field of empirical evidence. It defines caring and related behaviours that help nurses meet patient expectations. In this review, effective communication skills; spending quality time with the patients; relieving their anxiety by explaining the treatment plan; and making patients independent by involving them in planning their care, decision making, and self-care management were important. In this review, deficits in knowledge and training requirements of both nurses and student nurses have been identified. Nurse educators should nurture and empower the student nurses to internalise commitment and consistency in their caring behaviours [8]. The student nurses can learn caring concepts in the classroom settings and practice those concepts in the skills lab and clinical areas [26, 73, 74]. Studies have shown that education can help individuals to develop more caring attitude towards the patients [75, 76]. For nurse leaders, these review findings will bring insight to those who develop nursing policies to ensure that caring behaviours are incorporated into guidance and job descriptions for nurses. There is scant evidence of the impact on caring behaviours of patient outcomes, and more research into this specific area would be useful.

## Conclusion and recommendations

Using a mixed methods review, this paper provides a comprehensive synthesis of recent literature exploring the perception of caring behavior among patients, nurses, and nursing students. The majority of the studies included in this review highlighted the importance of physical and expressive care. However, the emotional and expressive aspects of care appeared to be less frequently discussed. Preliminary evidence was identified that suggests that nurse educators could focus more on the expressive aspects of care in the education of nursing students. For nurse leaders, the review findings will bring insight to those who develop nursing policies to ensure that caring behaviours are incorporated into guidelines and job descriptions for registered nurses. Nursing educators and hospital administrators should design interventions that strengthen both technical and expressive caring behaviours, ensuring that nurses balance clinical competence with relational and emotional aspects of care. Nursing education programs should integrate experiential learning and reflective practices that promote empathy, communication skills, and patient-centred care, preparing students to meet diverse patient needs effectively. Healthcare organizations should foster a supportive environment that recognizes, rewards, and monitors caring behaviours, and ensures adequate staffing and resources to allow nurses to provide holistic care. Conduct longitudinal studies to assess the impact of caring behaviours on patient outcomes, satisfaction, and nurse well-being. Explore cultural, contextual, and organizational factors influencing perceptions of caring in diverse healthcare settings. Develop and validate standardized tools for measuring both technical and expressive aspects of caring.

### Strengths and limitations

This review has a number of strengths enabling an in-depth insight into the perceptions of caring behaviours among patients, nurses, and nursing students. This review followed the Joanna Briggs mixed methods systematic review approach [20]. PRISMA [19] was used to report the process of the selection of the articles. All three reviewers screened each title and abstract of the selected articles, and any discrepancies about the inclusion of the articles were resolved. One reviewer (lead author) independently extracted the data, performed the analyses, and generated the themes. The other two co-authors reviewed the extracted data, analysis, and themes. A mixed methods appraisal tool (MMAT) was used to appraise the methodological quality of the studies to help assess quality standards for differing research designs [61].

A number of limitations of this review include the exclusion of the studies focusing on the perceptions of caring behaviours by the participants working in emergency departments, palliative care, community, and primary care, the selection of studies in the English language only and excluding the perceptions of family members and other health care staff. Additionally, there is a greater chance that relevant studies may have been overlooked because the searches were restricted to electronic databases and reference lists of qualifying studies [77]. Therefore, some potentially significant contributions in this area may have been missed.

A mixed-methods systematic review may be limited by the heterogeneity of included studies, including differences in study design, sample characteristics, settings, and measurement tools, which can complicate synthesis and comparison. Additionally, integrating quantitative and qualitative findings can be challenging due to differences in data type and reporting, and some contextual nuances from individual studies may be lost during aggregation. Finally, the conclusions are dependent on the quality and completeness of the included studies, limiting the generalizability of the findings.

## Electronic Supplementary Material

Below is the link to the electronic supplementary material.

Supplementary Material 1

Supplementary Material 2

Supplementary Material 3

## Data Availability

Data is presented in the main manuscript and additional supporting files.
