# Supplementary material for: Perceptions of caring behaviours among patients, nurses, nursing students: mixed methods systematic review
Source: BMC Nurs. 2026 May 9;25:561. doi: 10.1186/s12912-026-04600-4 (PMC13292442; doi:10.1186/s12912-026-04600-4)
Supplement: Supplementary file 1 — Supplementary Material 1 [file 12912_2026_4600_MOESM1_ESM.docx]

## Assessment of methodological quality

|  | **Screening questions**  **(for all types)** | | **Qualitative** | | | | |
| --- | --- | --- | --- | --- | --- | --- | --- |
| **Author/ Year / Country** | **Are there clear research questions?** | **Do the collected data allow to address the research questions?** | **Is the qualitative approach appropriate to answer the research question?** | **Are the qualitative data collection methods adequate to address the research question?** | **Are the findings adequately derived from the data?** | **Is the interpretation of results sufficiently substantiated by data?** | **Is there coherence between qualitative data sources, collection, analysis and interpretation?** |
| (Dobrowolska and Palese, 2016)/ Eastern Region of Poland | Y | Y | Y | Y | Y | Y | Y |
| (Ambrosi et al., 2021)/Italy | Y | Y | Y | Y | Y | Y | Y |
| (Petrou et al., 2017)/ Cyprus-Italy | Y | Y | Y | Y | Y | Y | Y |
| (Jill et al., 2015)/United Kingdom | Y | Y | Y | Y | Y | Y | Y |
| (Kalfoss et al., 2017 )/Oslo | Y | Y | Y | Y | Y | Y | Y |
| (Canzan et al., 2014)/Italy | Y | Y | Y | Y | Y | Y | Y |
| (Modic et al., 2014)/Midwest | Y | Y | Y | Y | Y | Y | Y |
| (Coughlin, 2013)/United States(Northeast) | Y | Y | Y | Y | Y | Y | Y |
| (Rahman et al., 2019)/Pakistan | Y | Y | Study design was not mentioned. However, a deductive approach was used to analyse the data | Y | Y | Y | Y |
| (Marshall et al., 2012)/South Australia | Y | Y | Y | Y | Y | Y | Y |
| (Mako et al., 2016)/Sweden (South) | Y | Y | Y | Y | Y | Y | Y |
| (Esmaeili et al., 2016)/Tehran | Y | Y | Y | Y | Y | Y | Y |
| (Sundus and Younas, 2020)/Pakistan | Y | Y | Y | Y | Y | Y | Y |
| (Andersson et al., 2015)/Sweden | Y | Y | Y | Y | Y | Y | Y |
| (Tsai and Wang, 2015)/Taiwan (Southern) | Y | Y | Y | Y | Y | Y | Y |
| (Cheruiyot and Brysiewicz, 2019)/ South Africa | Y | Y | Y | Y | Y | Y | Y |
| (Jardien-Baboo et al., 2016)/South Africa | Y | Y | Y | Y | Y | Y | Y |
| (Costello, 2017)/Boston | Y | Y | Y | Y | Y | Y | Y |
| (Pearcey, 2010a)/United Kingdom | Y | Y | Y | Y | Y | Y | Y |
| **Quantitative** | **Are there clear research questions?** | **Do the collected data allow to address the research questions?** | **Is the sampling strategy relevant to address the research question?** | **Is the sample representative of the target population?** | **Are the measurements appropriate?** | **Is the risk of nonresponse bias low** | **Is the statistical analysis appropriate to answer the research question?** |
| (Fang et al., 2020)/ East Coast of China | Y | Y | All the nurses were selected | Y | Y | 78.5% response rate | Y |
| (Fenizia et al., 2020)/Italy | Y | Y | All undergraduate student nurses | May be representative with 03 sample size, research conducted at two Italian universities | Y | Not mentioned | Y |
| (Allari et al., 2022)/Middle east ( Jordan, Palestine, Saudi Arabia, the United Arab Emirates, Oman and Egypt) | Y | Y | Second-third-and fourth-year student nurses /Convenience | Y | Y | Not mentioned | Y |
| (Akansel et al., 2021)/Turkey | Y | Y | Sampling strategy was not mentioned | Y | Y | 92.8 % response rate | Y |
| (Ferri et al., 2020)/Italy | Y | Y | All students were selected | Y | Y | 89.2% response rate | Y |
| (Labrague, 2012)/ Catbalogan City, Philippines | Y | Y | Purposive Sampling | Y | Pretesting and reliability and validity of the tool were not mentioned | Response rate was not mentioned | **Y** |
| (Zamanzadeh et al., 2014)/ Tabriz and Urmia faculties of nursing | Y | Y | Convenience sampling was done | Y | Questions were not pretested prior for the data collection/ reliability and validity of the tool were not mentioned | Response rate was not mentioned | **Y** |
| (Mlinar, 2010)/Slovenian | Y | Y | Collected the data from all the student nurses | Y | Questions were not pretested prior for the data collection. However, validity/ reliability were mentioned from the previous literature | Mentioned about the response rate, 76.6% with no justification mentioned | Y |
| (Labrague. et al., 2017)/Philippines Greece, Nigeria, India | Y | Y | Convenience sampling was done | Y | Y | 500 students were invited to participate in the study and 467 responded (93.4%) | Y |
| (Aktas and Karabulut, 2017)/Turkey | Y | Y | All the student nurses were selected | Y | Reliability and validity, pretesting of the tool were not mentioned | Didn't discuss about the response rate that might affect the analysis of the result | Y |
| (Trinidad et al., 2019)/Universidad Europea de Madrid | Y | Y | All the student nurses were selected | Y | Determined validity and reliability from the previous study | Not mentioned about the low response rate | Y |
| (Li et al., 2016)/Taiwan | Y | Y | Y | Y | Y | Y | Y |
| (He et al., 2013)/China (Central, Southern, and Eastern) | Y | Y | Convenience sampling was done | Y | Y | The study didn't discuss about the response rate that might affect the result | Y |
| (Aupia et al., 2018)/Indonesia | Y | Y | Sampling strategy was not mentioned | Y | Y | Y | Y |
| (Papastavrou et al., 2012) /Six different European countries (Finland , Greece Cyprus, Czech Republic Hungary,  Italy) | Y | Y | Convenience sampling was done | Y | Y | The study didn't discuss about the response rate that might affect the result | Y |
| (Flynn, 2016)/UK | Y | Y | Probability Stratified sampling method was used. | Y | Y | Didn’t discuss about the response rate that might affect the result. However, it was mentioned that 100 questionnaires were distributed, 50 each to the participants and researcher received  57 completed questionnaires | Y |
| (Omari et al., 2013)/ Jordan | Y | Y | Convenience | Y | Y | Didn't discuss about the response rate that might affect the result | Y |
| (Kiliç and Öztunç, 2015)/Turkey | Y | Y | All patients and nurses | Y | Y | Didn't discuss about the response rate that might affect the result | Y |
| (Roulin et al., 2020)/Geneva, Switzerland | Y | Y | Convenience sampling was done | Y | Cronbach alpha coefficient varies between .92 and .95 from the previous study. However, validity was not done and reliability .90 was mentioned. | Patients (85%) response rate The main reason of drop out was fatigue | Y |
| (Edvardsson et al., 2017)/Australia | Y | Y | Consecutive sampling strategy was used | Y | Not mentioned about the validity and reliability of the tool. Pilot testing of the tool was not done | Didn't discuss about the response rate that might affect the result | Y |
| (Merrill et al., 2012)/ghana | Y | Y | Convenience sampling was done. | Y | Questionnaire was not pretested prior to the data collection. However the validity/ reliability were mentioned | Didn't discuss about the response rate that might affect the result | Y |
| (Afaya et al., 2017)/Ghana | Y | Y | Probability Systematic sampling was done | Y | Not mentioned about the validity and reliability of the tool. Pilot testing of the tool was not done | A total of 200 questionnaires were distributed, out of that, a total of 183 questionnaires were recovered and considered for the analysis Response rate 91.5%. |  |
| (Youssef et al., 2013) /Taif City | Y | Y | Convenience sampling was used | Y | Y | Didn't discuss about the response rate that might affect the result | Y |
| Mixed Methods (Triangulation) | **Are there clear research questions?** | **Do the collected data allow to address the research questions?** | **Is there an adequate rationale for using a mixed methods design to address the research question?** | **Are the different components of the study effectively integrated to answer the research question?** | **Are the outputs of the integration of qualitative and quantitative components adequately interpreted?** | **Are divergences and inconsistencies between quantitative and qualitative results adequately addressed?** | **Do the different components of the study adhere to the quality criteria of each tradition of the methods involved?** |
| (Thomas et al., 2019) | Y | Y | Rational of choosing mixed methods design was not shared | Interrelation of quantitative and qualitative studies to form a complete picture | Meta-inferences done by interpreting qualitative and quantitative findings | Discrepancies in the findings were explained | Validity of the tool was not measured  Reliability Cronbach alpha .89 and .93 for the first and second survey  Rationale for choosing Mixed- Methods design was not mentioned.  Rationale for exclusion of participants was not mentioned  Non probability sampling techniques (Convenient) was used which does not represent the target population  The tool is justified and appropriate for answering the research question  Conceptual definition of each variable was not mentioned.  Any drop out/ withdrawal of the participants was not mentioned  Confounding variables can distort the interpretation of findings. However, this study didn’t mention about the confounding bias. |

## Data extraction

|  | | | | | |
| --- | --- | --- | --- | --- | --- |
| **Author/Country** | **Aim** | **Design** | **Setting/Sample/Sampling/Data Collection** | **Finding** | **Limitation** |
| (Dobrowolska and Palese, 2016)/  Eastern Region of Poland | Student nurses perception about caring, its features and possible hurdles | Qualitative content analysis | Nursing Institution/(n=15) Polish Students of three years degree programme/ Purposive/ Narrations has been written by the first year nursing students before and after their clinical experience./ Text‐diaries | Expressive caring was more valued and appreciated by the student nurse than the instrumental caring.  “caring comes from within the individual reflecting an internal human need, as the desire to do good”  “Caring means going towards the extra miles”  Helping patients to perform basic daily activities such as feeding, bathing, toileting.  Administration of medication on time, “changing patients’ position, offer things such as blanket, keep the things within reach of the patients, providing backrub, making the surrounding neat and clean before leaving the room”  “Protecting patients from physical harm such as fall , ulcers, malnutrition”  Monitor the patients for their well being  Spending time with patients, listening to them  “Promoting patients independence by allowing them to make independent decision”  Caring for the spiritual needs of patients  **Constraint**  He/she can have family problems or simply lack knowledge on providing good care (mostly preclinical students).” “Expectation of being appreciated, “she is caring but sometimes the patient is ungrateful”  “Nurses do not know when they should stop caring and let the patient become independent, prepared for self-care”  “Lack of time; too many patients and too many duties, tiredness; lack of independence at work; and lack of a satisfying remuneration.” | The two different time periods have been selected for the data collection. This might affect the behaviours of the participants because of the theoretical and clinical exposure. Narrations have been collected by the faculty members which might be biased the results. Findings of the study can't be transferable because it has been conducted in one University. |
| (Ambrosi et al., 2021)/Italy | To explore perception regarding caring behaviour among student nurses. | Qualitative longitudinal design | Student nurses (II and III year) (n=24)/convenience/semi-structured interviews and observation | Student nurses and staff nurses reassured the patients by touching them  Empathetic behaviour towards patients  Establishing trusting relationships with the patients was considered by all three- year students  Students in the second and third years of the nursing programme realised helping patients through continuity of care.  Students defined competency means correctly performing the skills in the first year. In contrast, in second and the third year, mastery of clinical skills and providing evidence-based practice was enhanced.  Keeping emotional distance from the patient was a concern for first-year nursing students. They perceived nurse can build a bond with their patient without becoming too attached to them. This may risk of creating a strong friendly relationship that may affect their ‘professional authority’ necessary to ‘maintain the nurse-patient relationship’  Students in their first year thought they had more time to spend with the patient.  The students in the second and third years understood the significance of this action but also the need to give each patient ‘the right time,’ which doesn't mean giving everyone the same amount of time but rather giving each patient the individualised amount of time they require and attending to all of their needs in a way that makes the patient feel at ease and at peace. | Study cannot be transferable because it has been conducted in one University. |
| (Petrou et al., 2017)/ Cyprus-Italy | Student nurses perception of caring | Qualitative approach | Nursing Department of a Cypriot University.  Students (n=122)  Questionnaires (open ended) | Helping patients to perform basic daily activities such as feeding, bathing, toileting.  Hold the patients’ hand and touch their arm and forehead for providing psychological support | Study conducted in one institution, findings may not be transferable. |
| (Phillips et al., 2015)/United Kingdom | Student nurses beliefs and values about caring at the initiation of the nursing programme | Longitudinal qualitative study | Nursing Institution/ (n=36 ) Undergraduate pre-registration nursing students from the two discrete programmes  (Advanced Diploma and BSc (Honours)/ Purposive/Semi-structured interviews | “Pharmacological intervention, medication and non-pharmacological actions ‘touching’ and ‘changing the patient’s position’ to relieve symptoms” | Lack of description about the rigour/ or trust worthiness of the study findings. |
| (Kalfoss and Owe, 2017)/Oslo | Explored concept of professional care from student nurses | Exploratory qualitative study/ The theoretical framework was based on Watson’s Human Caring | Nursing Institution/(n=31) Post bachelor students in Cancer Nursing, Nephrology Nursing, Pastoral Counselling, Public Health Nursing, and Masters’ students in Community Health Nursing /Purposive/ Focus group discussion | Respect for the uniqueness of the patients  Patients could keep a sense of autonomy and self-respect by being encouraged to make decisions. However, nurses cannot accept their decision when it causes risk or danger to their quality of life and health  Seeing for the whole person and not disease. One sees the human being in the patient  Caring means working in collaboration and delegating the responsibility to colleagues. This could make things go more smoothly.  Maintain confidentiality  “Not using empty words like stating “everything will be okay”, and not giving false hope.” | Collection of the data from a single setting. The findings may not be transferable to the target population. |
| (Canzan et al., 2014)/Italy | Comparison of the perceptions of caring from nurses and patients | Qualitative descriptive study | Hospital setting (gerontological department/(n=40)  (20) nurses and (20) patients/Purposive/  Semi‐structured interview | Invisible caring  Reflection and critical thinking of patients problems  Nurses need to be competent by using their clinical skills in reducing risks to the patients and preventing them from an emergency situations by recognising the signs and symptoms of an emergency situation  “nurse who remembered his/her  need and kept a promise: “When the nurses tell you ‘I  “Patients appreciated nurses who had positive attitudes and were cheerful, with them”  Patients has right to make independent decision  “The continuous provision of clear  information was valued by patients because it allowed them to participate in decision-making. | The trust worthiness of the data was not mentioned. |
| (Modic et al., 2014)/Midwest | Diabetic patients and nurses perceptions of caring behaviour | Qualitative descriptive study/survey | Acute care setting/(n=118)  (64) Nurses and (54) patients with diabetes/ Convenience/Survey/ open ended question. | Nurses stated that she listened to the patients when they were not cleared about the prescribed medication or its dosage.  “Thirty-four patients (62%) stated, nurses providing information about their blood sugar results and informed of upcoming tests and procedures”  The six (11%) patients who experienced hypoglycemia received prompt care from nurses  Teaching was the most prevalent caring behaviour identified by 41 (76%) of nurses. | Trust worthiness of the data was not mentioned. |
| (Coughlin, 2013)/ United States (Northeast) | Perceptions of nurses and patients about care during key events of the hospitalisation (admission, transfer to the operation room and preparation for discharge | Ethnographic methodology | Hospital setting/(n=12)  (10) patients and (2) nurses/sampling strategy not mentioned/Participant observation and unstructured interview | Laughing and talking with them  patients were uncomfortable by having hard bed, noise, and tasteless food. Whereas nurses perceived, pillows were not in good condition, ward was noisy at night due to noisy equipment, food was not appropriate for cardiac patients  Patients in the surgical unit observed nurses responded late and they expected response time should be improved  It was observed by the researcher that the patient seldom required to use their call bells because nurse anticipated their needs on time | A very small number of sample and the participants from one setting, the findings may not be transferable to the target population. |
| (Rahman et al., 2019)/Pakistan 2019 | Health care providers and patients. Perceptions regarding best practices in patient‐centred care (PCC). Identified the similarities of perspectives between both the groups | Qualitative descriptive study | Tertiary care hospital (Orthopaedic  Department)/ (n=36)  (18) Health care providers (nurses, consultant doctors,  Residents, radiologists, and physiotherapists) and (18) patients/Purposive sampling/ six focus group interviews (FGIs) | Patients and nurses agreed that empathetic behaviour promotes a collaborative relationship with them. Both groups agreed that providers spend time to counsel their patient and relieved their doubts and fears. In decision making, family involvement was also an important factor for patients. | Patients were getting free‐of‐cost services from the hospital, responses may be biased because of this facility. Results from the private sector may not be transferable to the public sector. |
| (Marshall et al., 2012)/ South Australia | Understanding of patients about patient‐centred care and identified the relationship of it with the existing literature. | Qualitative phenomenologic | Metropolitan Hospital (surgical unit)/(n=10 ) Patients/Purposive/ Interpersonal interviews | Nurses ensured to come on time when they called them and fulfil their needs  The use of humour by staff nurses as a coping mechanism to fulfill emotional needs of their patients. According patient making jokes about various topics can help them stay positive and divert their mind  The patients in surgical unit complained about the tasteless food and variation in room temperature  Participants identified the need to see other patients and do paperwork as major barriers to the staff providing the best care they could  Despite their lack of medical knowledge, they are nonetheless experts in their illnesses. They should be regarded and respected rather than just a “number | Trust worthiness of the data was missed. One single department and site may affect the transferability |
| (Mako et al., 2016)/Sweden (South) | Patients’ meaning of good care | Constructivist grounded theory | Surgical department/ (n=13 ) patients/ Theoretical/ Face to face interview | Ensure patients feel safe  Reducing patient’s anxiety while performing invasive procedure  Act of listening to people  Patients has right to make independent decision | A small number of participants, transferability is questionable. |
| (Esmaeili et al., 2016)/Tehran | Cardiac patients perceptions of patient-centred care | Descriptive qualitative study | Cardiac Unit/(n=18) cardiac patients/ Purposive/Semi-structured interviews | “Protecting patients from physical harm ( fall , ulcers, malnutrition)”  Patients who experience poor communication feel disregarded and neglected. They believed that treating patients with respect in the beginning and throughout their hospitalisation gives them a great deal of confidence and comfort in the stressful environment. Patients expected nurses to just greet them with a smile.  Patient believed that making decisions without taking patients' opinions into account, it showed disrespecting or ignoring patients' expectations | A small number of participants, transferability is questionable |
| (Andersson et al., 2015)/Sweden | Registered nurses perceptions about the caring concept | Qualitative design phenomenog raphic | Coronary Care Unit /(n=16) Nurse/Purposive/ Interview | Nurses checked the vital signs and promptly recognised the changes in the health condition of the patients  Evaluating the effects of different types of treatment | A small number of participants, transferability is questionable |
| (Tsai and Wang, 2015)/Taiwan (Southern) | Perceptions of registered nurses about the caring behaviour | Qualitative study | Hospital setting/(n=58) Nurse/Convenience sampling/Semi-structured interview | “Providing physical comfort patient felt higher satisfaction”  Reduce patients’ anxiety while performing invasive procedure  Possessing the practical competences is required to perform the procedure correctly. This behaviour may ensure the patients that they are safe and prevent them from physical pain or anxiety. | Trust worthiness of the data and the pilot testing of the interview guide was not mentioned. Involved one hospital and the participants were only females, the study findings may not be transferable. |
| (Cheruiyot and Brysiewicz, 2019)/South Africa | Perceptions of caring and uncaring nursing encounters | Qualitative descriptive stud | Inpatient (Rehabilitation setting)/(n=21) Nurses/Purposive/Semi-structured individual interview | “going the extra mile,”  **Uncaring behaviour**  Patients from the rehabilitation department might occasionally be demanding, annoying, and irritated nurses. Patients were aggressive with nurses as well, shouting at them. The nurse thought the patient did not like her. This affected her to demonstrate caring behaviour  Nurses were not doing enough to safeguard the patients and keep them safe. When nurses failed to promote patient safety, they were unhappy.  Nurse claimed that they did not ignore the patients consciously. They might busy with other patient.  Motivating the patients to keep a fighting spirit, they assisted the patients in coping with their impairments and overcoming the difficulties. By consistently reassuring the patients that they were making adequate progress even when they weren't, they created and maintained hope in their lives. | Limitation of the findings to be transferable to the culturally diverse patients from rehabilitative department. |
| (Jardien-Baboo et al., 2016)/South Africa | Perceptions about patient-centred care from the professional.  Enabling and inhibiting factors to patient centered care. | Qualitative descriptive | Public hospital(n=40) Nurses/Purposive/ semi-structured interviews | Their perception that the patient should be seen as a  human being with various components such as psychological, physical, social, emotional and spiritual aspects which impact on an individual's health was evident.  **Obstacle patient-centered care:** ‘a lack of adequate resources’, ‘increased administrative work’  Caring behaviour  Gestures such as smiling and the manner in which a patient is addressed  The tone which the nurse uses should be respectful and not loud  **Enabling environment**  Both nurses and nurse managers emphasised the importance of positive working environment.  The need for continuous in-service education and training on  different topics was mentioned by all the participants  **Resources**  Participants voiced their frustrations with regard to the non-appointment of staff in the face of staff shortages, the resultant increased workload, as well as limited equipment,  Displays a lack of nursing ethos. The harsh manner in which some nurses communicate with patients causes patients to feel upset and afraid and ultimately, they are less likely to communicate their needs | A study has been conducted in the public hospitals. Findings could not be transferable to private hospitals. |
| (Costello, 2017)/Boston | Identified the characteristics and behaviours from nurses who were identified by the patient as a best nurses | Qualitative Study | Medical Surgical unit/(n=9) nurses/Sampling not mentioned/ Focus group | Caring for the spiritual needs of patients  “knowing the patient” is important. They emphasised the significance of truly being present for patients rather than simply rushing in to finish tasks.  “Nurses described praying with patients of faiths different than their own because they believed that this would make the patients feel better.”  Patients whose religious beliefs differ from the nurses received spiritual support from them. They prayed with patients who practised other religions because they thought that would help the patients to feel better | Trust worthiness of the data was not mentioned |
| (Pearcey, 2010)/United kingdom | Opinion of qualified nurses about the central values in clinical nursing | Grounded theory approach/ | Hospital setting (n=25) qualified nurses/Sampling not mentioned/Semi structured interviews | This was particularly obvious when some interviewees indicated they were busy doing physical care but had no time for emotional care.  The majority thought students need to see caring behaviour in the clinical area but also held the view that it was something innate that the students needed to bring with them. | Trust worthiness of the data was not mentioned |
| (Sundus and Younas, 2020)/ Pakistan | Patients perspective of caring behaviours of male nurses | Descriptive qualitative study drawn from a larger convergent mixed-methods | 14 Medical Surgical Departments of three private hospitals in Islamabad, Pakistan/ Semi‐structured interviews/ purposive/ Patients (n= 15) | “Strong knowledge about different conditions of patients with different diseases”  Nurses advocating on his/her behalf to the rest of the team  Apologising for late response to call for care | Broader transferability of the findings is limited because of the secondary data from the previous study. |
| (Fang et al., 2020)/ East Coast of China | To explore perceptions of caring based on ‘CARE model’ from nurses. To provide practical guidelines for nurses to improve their behaviour. | Cross-sectional survey | Nurses working in the following units of the internal medicine department, Endocrinology, Respiratory, Cardiology, Gastroenterology, Pain, Neurology, Nephrology, Oncology, and Hematology/ (n=157) four dimensions of CARE Model | Competence (68.79%), Altruism (73.25%), Responsibility (86.62%), Empathy (81.53%).  Narrative response  “hands-on ability Nurses need to work efficiently and provide safe and accurate care and avoid medical error  Nurses believed helping the other colleagues in the unit within their capacity to help patients is important | Study conducted in one university, results may not be transferable. |
| (Akansel et al., 2021)/Turkey | To investigate nurses perception on caring activities | Descriptive study design | Nurses working in one university hospital/ (n=260)/25-item- Caring Dimensions Inventory | Explain the clinical procedure to the patient. Observe the effects of medication.  Patients have a right to be treated in accordance with their cultural beliefs. Involving patients in their care should be considered as important caring activities in nursing. However, in this study, these behaviour was least consider by nurses due to high patients’ ratio. | Study conducted in one University, results may not be generalised. |
| (Allari et al., 2022)/ Middle East | To explore and compare perception of undergraduate student nurses about caring | Cross-sectional-descriptive, comparative design | Nursing students (n= 1,582)/ convenience/Caring Dimensions Inventory | Nurses considered privacy for a patient (M=4.86 SD=0.519) Students checked the vital signs and promptly recognised the changes in the health condition of the patients (M = 4.79, SD = 0.61) | use of self-report surveys, which might have caused participants to give socially acceptable answers. The study's generalisability was further restricted by the use of networking and snowball sampling. |
| (Fenizia et al., 2020)/ Italy | Analyse the variations in caring behaviour among student nurses during the academic year. | Descriptive longitudinal study | Undergraduate student nurse (II and III year) (n=103)/ Italian Caring Behaviors Inventory questionnaire | Doing the task competently was also considered by second- and third- year students  Second- and third- year students perceived least consider in responding to patients individual needs | Sample size should have been increased  Participants should have been selected from more universities |
| (Labrague, 2012)/ Catbalogan City, Philippines | Perception of patients towards caring competencies of Level IV students | Descriptive research | Different clinical units of Samar Provincial Hospital/ (n= 174 patients)/Purposive Sampling/CBA -63 Caring Behaviour Assessment Tool | Highest rated by patients in “Knowledge and skills ”subscale ‘Know how to give shots, IVs’, “Respectful” ‘Kind and considerate’, “Assurance” ‘Give me treatments and medications on time; and least rated in “Assurance”, ‘Talk to me about my life outside the hospital’, “Trusting relationship” ‘Ask me what I like to be called’, ‘Introduce themselves to me “Teaching and learning“ ‘Tell me what to expect during the day’ | Response rate, representation of the sample, pretesting and reliability and validity of the tool were not mentioned. |
| (Zamanzadeh et al., 2014)/Tabriz and Urmia faculties of nursing | Perceptions of student nurses toward caring behaviour | Cross-sectional | Nursing School/ (n=230) All first and fourth-year nursing students/ Convenience sampling/Larson's Caring Questionnaire Caring Assessment Questionnaire (Care-Q) | Both groups rated higher score in the Subscale “monitors and follows through" “ to demonstrate professional competency and be assured that nursing actions delegated to others were completed (e.g., to know when to call the doctor, etc.)” “to give a quick response to the patients' call” and rated lower score in "anticipates" “to anticipate the changes in patient’s situation and take anticipatory actions (e.g., is perceptive of the patients' needs and plans or acts accordingly, etc.)”and "trusting relationships" “to convey a sense of commitment and understanding to patients (e.g., when with a patient, to concentrate only on that one patient, etc.)”  Fourth year students rated higher than the first-year in subscale "explains and facilitates teaching, “clarifying, and advocating (e.g., to teach the patient how to care himself/herself whenever possible, etc.)" | Representation of the sample and validity reliability of the tool were not mentioned |
| (Ferri et al., 2020)/ Italy | Perceptions of caring behaviour by student nurses | Three-cohort observational study | Nursing School/(n=331) All the students selected / CBI-24 | “Responding to individual needs” and “Being with” highest rated among the first year students. At the end of the first year, students were able to demonstrate expressive care. Instrumental care developed in the second and third year.  Students encouraging patients to call them in case of problems (5.30 (0.8), 5.52 (0.9), and 5.70 (0.7, respectively) and responding them promptly (4.80 (0.9), 5.05 (1.0), and 5.28 (0.8, respectively). | Study conducted in one University, results may not be generalised. |
| (Mlinar, 2010)/Slovenian Rou | Identification of the significant differences in the mean scores among the first-year and third-year student nurses. | Survey design | Nursing School/(n=166) first-year and third-year nursing students/Sampling not mentioned/Watson’s Transpersonal Caring Theory instrument | Third-year students scored higher in perceptions of caring behaviour as compared to the first-year students.  Among the nursing students the most important caring behaviours under the subscales of, “Respectful”: ‘being respectful to patients’, “Teaching / learning”: ‘teaching them’ and “Trusting Relationship”: ‘relationships with patients’ | Study conducted in one university , result may not be generalised. |
| (Labrague et al., 2017)/ Philippines Greece, Nigeria, India | Explored about the caring behaviours from student nurses in the four countries | Descriptive comparative survey design | Nursing School(n=467) Nursing Students/ Convenient sample/Jean Watson’s theory of human caring based on Watson’s theory of 10 carative factors | Highest rated by the students In the subscale of “assurance of human presence” ‘gives patient treatments and meds on time’ (4.961 ± 1.054).  Nurses from the coronary care unit were less competent in skills such as giving injections, administering intravenous medication, and managing and handling equipment (4.630 ± 1.348)  and ‘allows the patient to express feelings about his or her disease and treatment’, in the subscale of “Trusting and relationship” ‘treats patient information confidential’  The students rated lowest scores to the ‘empathetic with the patient’ ‘spends time with the patient’ ‘and in knowledge and skills subscale: ‘knows how to give shots, IVs’ | Use of the convenience sample may limit the generalisability of the findings. |
| (Aktas and Karabulut, 2017)/Turkey | Correlation of the undergraduate student nurses 'professional values and their caring behaviour. | Cross-sectional descriptive survey design | Nursing School/(n=351) First- Fourth year Undergraduate students/Sampling not mentioned/Nursing Professional Value Scale and Caring  Assessment Questionnaire Care-Q | The professional value and care behaviour were found to be lower in the students in the year II than those in the I, III, and IV year. | Study was done on one nursing institution which limits the generalisability of the findings of the study. |
| (Trinidad et al., 2019)/Universidad Europea de Madrid | Undergraduate student nurses perception about caring and identified any differences in the behaviour among them. | Cross-sectional design | Nursing School/(n=321) Undergraduate nursing students/Sampling not mentioned/Caring Dimensions Inventory (CDI-25) | The most important caring behaviours perceived by the students in the subscale of “Respect”: ‘Providing privacy for a patient’ (M=4.86, SD=0.405) and in the subscale of “Attentiveness”: ‘Listening to a patient’, and the least ranked in “Trusting relationship”: ‘sharing your personal problems with a patient’. First year students consider technical / Instrumental care, however, for third and fourth, psychosocial care is most important. | Study was done on one nursing institution which limits the generalisability of the findings of the study. |
| (Li et al., 2016)/Taiwan | Comparison of the views about caring behaviors among students and registered nurses | Cross‐sectional study | Nursing School and Clinical Setting/ ( n=647) Participants from the three programmes (330) nursing students: Five-year ADN programme, the two-year and four-year baccalaureate degree of nursing programmes.  (317) registered nurses.  from medical, surgical, obstetric, paediatric and intensive care units/ Convenience/Traditional Chinese caring behaviours scale (CBS) | The most important caring behaviour is ‘knowing the patient’, Least important ‘advocating for the patient’, respect the patient's and ‘family's best interests, voicing for them’. | This study took place in a single university, therefore, findings may not be generalised. Proportion of the male was less in order to compare the gender differences. |
| (He et al., 2013)/ China (Central, Southern, and Eastern) | Comparison of the perspectives among nurses and patients about caring behaviour | Descriptive comparative survey | Five hospitals in southern, central and eastern China. Each three medical surgical unit / (n=1220) Patients and nurses/Convenient sampling/Caring Behaviours Inventory-24. | Both the groups perceived knowledge and skills at the highest level.  nurse administered the medication on time ( M=4.65).  patients and nurses indicated that encouraging patients to call them in case of problems (4.70 and 5.45, respectively). | Convenience sampling was done which limits the generalisability of the study findings. |
| (Aupia et al., 2018)/Indonesia | Comparison of the perceptions of caring behaviours among nurses, patients and student nurses. Explored the correlation between demographic variables and the caring. perception | Descriptive comparative study | One Hospital and one nursing school/(n=159)  (53) nurses, (53) patients and (53) student /Sampling not mentioned/Caring Behaviour Inventory-42 | Students with clinical experiences (8-week) scored higher in the caring aspect connectedness (t=3.50, p< 0.05) as compared to students with (4-week) of experiences. | The study was conducted in one hospital and school of nursing, therefore, findings may not be generalised. |
| (Papastavrou et al., 2012)/Six different European countries (Finland , Greece Cyprus, Czech Republic Hungary, Italy) | Perceptions of patients and nurses about caring behaviours. | Descriptive comparative survey | Surgical Unit/ (n = 2854)  (1659)Surgical patients and (1195) nurses/Convenient sampling/Caring Behaviours Inventory‐24 | According to nurses and patients, most important caring behaviour is knowledge and skills.  Lowest rated listening to their concerns  Patients perceived that nurses did not consider being empathetic as compared to nurses' perceptions. Trusting relationship is rated lowest by patients | Use of convenience sampling which unable to generalise the findings. |
| (Flynn, 2016)/UK | Perceptions of caring from both patients and healthcare professionals | Descriptive study | Acute hospital trauma ward of the orthopaedic department/(n= 83) (30 ) Patients and (53) Healthcare Professionals (doctors, nurses, physiotherapists and occupational therapists/Probability Stratified sampling/Caring Behaviours Inventory (CBI) Theory of transpersonal caring | Nurses and patients rated higher score in: “Attentive” subscale ‘attentively listening to the patient’ and ‘watching over the patient’. “Knowledge and skills”, ‘demonstrating professional knowledge and skill’ and ‘giving good physical care’. “Connectedness” patients rated lowest ‘touching the patient to communicate caring’ | Participants from the one district general hospital and the low response rate may confine generalisability. |
| (Omari et al., 2013)/Jordan | Perception of patients and nurses about caring behaviours.  Comparison of perceptions among both the groups | Descriptive comparative design | Coronary care unit (n=210) (150) Patients and (60) nurses/ Convenience/ Caring Behaviour Assessment | Patients perceived caring behaviours (technical and physical care) as most important, however, nurses perceived teaching/ learning behaviours as most important.  Nurses from the coronary care setting clarified the queries raised by the patients (M=4.68), enquired about their understanding (M=4.32), helped them to plan a realistic goal for their health (M=4.30)  On instrumental caring behaviours such as knowing how to give injection, IV infusion, and how to handle procedural equipment were not considered the most important by nurses.  Nurses commented in this study that competency in skills is not considered as caring behaviour; however, it is a prerequisite for nursing. Furthermore, other nurses believed that explaining procedures to the patients is more important to demonstrate caring behaviour than competent skills.  Due to critical nature of the coronary disease, nurses in coronary care units believed that listening to the patients is the most important caring behaviour because patients were more at the risk of psychological distress, such as depression, anxiety, and mood swings, which may lead to further deterioration of heart function.  For Muslims, religion directs their actions and thoughts toward God. Patients' religious beliefs and practices are considered as spiritual needs. During their illness, Muslim patients often perform their spiritual religious practices, such as reading the Qur'an (Islam's holy book) and praying.  Students and patients from coronary care unit rated the nurses lowest in the trusting relationships.  Nurses from the coronary care setting clarified the queries raised by the patients, enquired about their understanding, helped them to plan a realistic goal for their health.  The nurses many a times may feel uneasy and inconvenient while providing care to their patients due to the undue visit of their family members. | Representation of the sample and response rate was not mentioned. |
| (Kiliç and Öztunç, 2015)/Turkey | Comparison of the perceptions of patients and nurses | Descriptive study | Surgical operation department (n=449)  (379) Patients and (70) nurses/Sampling not mentioned/CBI-24 (Caring Behaviours Inventory-24) | Patients rated lower score than the nurses in the subscale of: “knowledge and skill” and “being respectful” p<0.001 | Homogeneous ethnic and racial 100% Caucasian sample may not be representative to the target population. |
| (Edvardsson et al., 2017)/Australia | Association of patients' perspectives about the caring behaviour and person‐ centeredness with the outcome of quality care nursing. | Descriptive non‐experimental  correlational design | Tertiary acute‐care setting/Patients 210/consecutive sampling/Caring Behaviours Inventory, the Person-centred Climate Questionnaire, the SF-36 and the Distress thermometer | Patients reported that the quality of nursing care is due to effective communication, knowledgeable staff, timely assistance and environmental support, for example, neat and clean ward, feel like home | Use of consecutive sampling and single study site which affect generalisability. Exclusion of the patient with the severe illness or participants were unable to understand or speak English, consequently, the results may be valid for an English-speaking population |
| (Merrill et al., 2012)/Ghana | Patients perceptions of caring behaviours | Descriptive study | Medical- surgical ward (Trauma centre)/(n=105) Patients/Convenience/Caring Behaviours Inventory/1-to-1 interview | Patients rated the nurses highest in the subscales of “knowledge and skills” "Meeting the patient's stated and unstated needs," "Being confident with the patient," and “Assurance”:"Giving the patent’s treatments and medications on time." Nurses also involved patients in planning their care such as what they needed to get done on a particular day and develop a planner  Rated lowest scores in the subscales of “connectedness” "Touching the patient to communicate caring" | Questions were not pretested prior to the data collection. However, validity and reliability was mentioned from the previous literature. Study didn't mention about the response bias. |
| (Afaya et al., 2017)/Ghana | Patients perceptions about caring behaviour | Descriptive cross-sectional study | Medical- Surgical Ward(n=183) Patients/Probability Systematic sampling Caring Behaviours Inventory-24. | 89.1 % patients strongly agreed that nurses attentively listen to them. 87.4% respondent perceived they gave instructions and taught them.  91.8% of respondents perceived that nurses were competent in giving injection and administering IV medications.  90.2% indicated that nurses were having professional knowledge and skill.  In accordance with 91.2% of participants, nurses appeared concerned when attending to them.  The top ranked caring behaviours, according to 92.9% of respondents, involved nurses giving patients their treatment and medication on time. | The study didn’t discuss about the representation of the sample |
| (Youssef et al., 2013) /Taif City | Perceptions of caring behaviours from nurses | Quantitative descriptive correlational design | Medical-Surgical Nurse's/(n=90) /Convenience sample/Watson's Tanspersonal theory (CBA) | Nurses treat the patients as an individual and respect them.  ‘Be kind and considerate’ to be the most important caring behaviours (4.3±0.9) and help them how to achieve goals (4.3±5.2)  In Trusting relationship, nurses perceived the least important caring behaviours ‘Touch me when I need it for comfort’ ( 2.8±1.4) | Use of nonprobability convenience sampling, can't generalise the findings. |
| (Roulin et al., 2020)/Geneva, Switzerland | Describes and compares nurses' and admitted patients' perceptions of caring behaviours | Comparative descriptive design | Rehabilitation  Nurses (n=34)  Elderly (n=64)  Convenience  Watson Caring Nurse Patient Inventory-23 | Patients perceived nurses knew what to do in emergency situations and act quickly  Least important caring behaviour of nurses (Patients)  Nurse considered patients as an individual and not only interested in resolving health problems  Least important caring behaviour (Nurses)  Provided opportunity to the patients to take care of themselves  Give treatment and medication on time. | Sample size was not calculated  Convenience sampling instead random affects validity |
| (Thomas et al., 2019)/North Texas | Congruency of perceptions of nurse caring behaviour between patient and nurse. Determine patient perception changes over time. | Mixed Methods Triangulation Design | Long-term acute care hospital  Convenience  Patients (n=25)  Caring Assessment Tool (CAT-V) filled during first 1 to 2 weeks after admission and during the week of discharge  Nurse= (n=85) brief stories (patient and family encounter) | Patients and nurses considered nurses showing respect towards patients  The patients report higher satisfaction when nurses provide physical comfort by fulfilling their basic physical needs  Patients rated low scores to nurses for asking about patients’ knowledge of their illnesses.  Nurses described they informed them ‘about their illnesses, not asking about their knowledge of their illnesses.’  Patients rated high scores to nurses for ‘helping them feel comfortable or attending to their basic physical needs’  They discussed with the patients about care planning of the day.  The least caring behaviours were talked to the patients while providing care to them. | Non probability sampling techniques (Convenient) was used which does not represent the target population. |
