# Supplementary material for: Perceptions of caring behaviours among patients, nurses, nursing students: mixed methods systematic review
Source: BMC Nurs. 2026 May 9;25:561. doi: 10.1186/s12912-026-04600-4 (PMC13292442; doi:10.1186/s12912-026-04600-4)
Supplement: Supplementary file 2 — Supplementary Material 2 [file 12912_2026_4600_MOESM2_ESM.docx]

## Spider search strategy

**Question:**

1. What are the perceptions of the adult patients regarding caring behaviour of nurses admitted in the hospital?

S- **Sample**: adult, inpatient, acute care setting, hospitalisation, medical-surgical

P of I – **Phenomenon of Interest**: caring behaviour, compassion, compassionate care, attitude, hospital care, empathy, patient’s experience, patient’s satisfaction

D-**Design**: questionnaire, interview, focus group, observation

E- **Evaluation**: perception, patient’s perception, view, experience, perspective

R- **Research type**: qualitative, quantitative, mixed methods

**Search strategy:**

Individual and combined the terms with the Boolean operators (AND, OR and NOT)

**Limits applied to the search:**

Age: young to older adult

Year(s) of publication: 2009-2023

Language: English

**List of the databases:**

Medline, PsycINFO, Embase, CINAHL, Scopus and Web of Science

**Question:**

What are the perceptions of nurses regarding caring behaviour working in the hospital?

S- **Sample**: nurse, inpatient, acute care setting, hospital, medical- surgical

P of I – **Phenomenon of Interest**: caring behaviour, compassion, compassionate care, attitude, hospital care, empathy, attitude of health personnel

D-**Design**: questionnaire, interview, focus group, observation

E- **Evaluation**: perception, nurses perception, view, experience, perspective

R- **Research type**: qualitative, quantitative, mixed method

**Search strategy:**

Individual and combined the terms with the Boolean operators (AND, OR and NOT)

**Limits applied to the search:**

Year(s) of publication: 2009-2023

Language: English

**List the database:**

Medline, PsycINFO, Embase, CINAHL, Scopus and Web of Science

**Question:**

1. What are the perceptions of student nurses regarding caring behaviour?

S- **Sample**: student nurses, inpatient, medical-surgical. Acute, setting, health care

P of I – **Phenomenon of Interest**: caring behaviour, compassion, compassionate care, attitude, hospital care, empathy, experience,

D-**Design**: questionnaire, interview, focus group, case study, observation

E- **Evaluation**: perception, view, experience, perspective

R- **Research type**: qualitative, quantitative, mixed method

**Search strategy:**

Individual and combined the terms with the Boolean operators (AND, OR and NOT)

**Limits applied to search:**

Year(s) of publication: 2009-2023

Language: English

**List of the database**

Medline, PsycINFO, Embase, CINAHL, Scopus and Web of Science

## Search strategy

1 (nurse* adj (view* or opinion* or attitude* or concern* or belief* or feeling* or idea* or perce* or perspective* or esxpectation* or preference* or need* or satisfaction)).tw. (10555)

2 "attitude of health personnel"/ (52329)

3 caregivers/ [psychology] (51878)

4 or/1-3 [ nurses perception] (111635)

5 empathy/ (21901)

6 empathy.tw. (12990)

7 compassion*.tw. (11764)

8 kindness.tw. (976)

9 (caring adj5 behavio?r*).tw. (695)

10 or/5-9 [caring behaviour] (35413)

11 4 and 10 [nurses perception and caring behaviour ] (2934)

12 (acute adj (setting* or hospital* or care or healthcare)).tw. (36532)

13 hospital care.tw. (9978)

14 exp hospitals/ (956307)

15 hospitalization/ (330763)

16 (hospitali?ation or hospitali?ed).tw. (304358)

17 Inpatients/ (126925)

18 inpatient*.tw. (151792)

19 medical surgical.tw. (7866)

20 or/12-19 [hospital] (1455076)

21 11 and 20 [nurses perception and caring behaviour and hospitals] (406)

22 limit 21 to (english language and yr="2009 - 2023") (204)

23 ((patient* or user*) adj3 (view* or opinion* or attitude* or concern* or belief* or feeling* or idea* or perce* or perspective* or experience* or expectation* or preference*OR need* or satisfaction or interaction*)).tw. (462588)

24 exp patient satisfaction/ (127244)

25 patients/ [psychology] (1042995)

26 or/23-25 [ patient experience] (1503810)

27 empathy/ (21901)

28 empathy.tw. (12990)

29 compassion*.tw. (11764)

30 kindness.tw. (976)

31 (caring adj5 behavio?r*).tw. (695)

32 or/27-31 [caring behaviour] (35413)

33 26 and 32 [ patient experience and caring behaviour] (5655)

34 (acute adj (setting* or hospital* or care or healthcare)).tw. (36532)

35 hospital care.tw. (9978)

36 exp hospitals/ (956307)

37 hospitalization/ (330763)

38 (hospitali?ation or hospitali?ed).tw. (304358)

39 Inpatients/ (126925)

40 inpatient*.tw. (151792)

41 medical surgical.tw. (7866)

42 or/34-41 [hospital] (1455076)

43 33 and 42 [ patient perception and caring behaviour and hospitals] (1232)

44 limit 43 to (english language and yr="2009 - 2023") (1008)

45 (student nurse* adj3 (view* or opinion* or attitude* or concern* or belief* or feeling* or idea* or perce* or perspective* or experience* or expectation* or preference* or need* or satisfaction)).tw. (610)

46 "attitude of health personnel"/ (52329)

47 caregivers/ [psychology] (65200)

48 or/45-47 [ nursing students perception] (117106)

49 empathy/ (21901)

50 empathy.tw. (12990)

51 compassion*.tw. (11764)

52 kindness.tw. (976)

53 (caring adj5 behavio?r*).tw. (695)

54 or/49-53 [caring behaviour] (35413)

55 48 and 54 [ nursing student perception and caring behaviour] (2890)

56 (acute adj (setting* or hospital* or care or healthcare)).tw. (36532)

57 hospital care.tw. (9978)

58 exp hospitals/ (956307)

59 hospitalization/ (330763)

60 (hospitali?ation or hospitali?ed).tw. (304358)

61 Inpatients/ (126925)

62 inpatient*.tw. (151792)

63 medical surgical.tw. (7866)

64 or/56-63 [hospital] (1455076)

65 55 and 64 [ nursing student perception and caring behaviour and hospitals] (391)

1. limit 65 to (english language and yr="2009 - 2023") (197)
