# Supplementary material for: Perceptions of caring behaviours among patients, nurses, nursing students: mixed methods systematic review
Source: BMC Nurs. 2026 May 9;25:561. doi: 10.1186/s12912-026-04600-4 (PMC13292442; doi:10.1186/s12912-026-04600-4)
Supplement: Supplementary file 3 — Supplementary Material 3 [file 12912_2026_4600_MOESM3_ESM.docx]

Thematic analysis of Qualitative Data

| **Codes** | **Descriptive themes** | **Analytical Themes** |
| --- | --- | --- |
| Reflection and critical thinking (Canzan et al., 2014) | Knowledge and Skills | Physical Care |
| Thinking about patient’s needs (Canzan et al., 2014) addressing patient’s needs (Pearcey, P., 2010; Fang et al., 2020)  “Nurses need to focus on the needs of the unit and patients regardless of personal gains or losses.” (Fang et al., 2020) |  |  |
| Evaluating the care being provided (Canzan et al., 2014) |  |  |
| “Strong knowledge about different conditions of patients with different diseases” (Canzan et al., 2014; Sundus and Younas, 2020; Fang et al., 2020) |  |  |
| Promptly recognised changes (signs and symptoms) (Canzan et al., 2014) |  |  |
| “Caring is about to find the peoples’ problem and solve it (Dobrowolska and Palese, 2016; Pearcey, P., 2010). |  |  |
| “Assessing vital signs and physiological changes”(Jill et al., 2015; Andersson et al., 2015) |  |  |
| “Understand the patient’s body language” (Andersson et al., 2015) |  |  |
| Updated knowledge and clinical skills (Kalfoss, M. et al., 2017 ; Andersson et al., 2015; Fang et al., 2020)  “hands-on ability and improve work efficiency”(Fang et al., 2020) |  |  |
| “Collecting data about the patients to form a basis for diagnosis, assessment and action” (Andersson et al., 2015) |  |  |
| “Pharmacological intervention, medication and non-pharmacological actions ‘touching’ and ‘changing the patient’s position’ to relieve symptoms” (Jill et al., 2015; Andersson et al., 2015). |  |  |
| Evaluating the effects of different types of treatment (Andersson et al., 2015) |  |  |
| Caring as the ‘extra’ or  ‘extra mile’(Dobrowolska and Palese, 2016; Kalfoss, M. et al., 2017 ; Amy et al., 2012; Pearcey, P., 2010) |  |  |
| “Providing physical comfort patient felt higher satisfaction” (Thomas et al., 2019; Tsai, Y.-C. and Wang, Y.-H., 2015) | Comfort |  |
| Helping to perform basic daily activities ( feeding, bathing, toileting) (Andersson et al., 2015; Canzan et al., 2014; Dobrowolska and Palese, 2016; Petrou et al., 2017; Thomas et al., 2019) |  |  |
| “Treat my body carefully” (Thomas et al., 2019) |  |  |
| “Ward and room environment comfortable” (Sundus and Younas, 2020) |  |  |
| “Administration of medication, changing patients’ position, offer things such as blanket, keep the things within reach of the patients, providing backrub, making the surrounding neat and clean before leaving the room” (Andersson et al., 2015; Canzan et al., 2014; Dobrowolska and Palese, 2016; Edvardsson et al., 2017; Omari et al., 2013; Phillips et al., 2015; Tsai, Y.-C. and Wang, Y.-H., 2015; Youssef et al., 2013) |  |  |
| Relieving physical pain (Petrou et al., 2017) |  |  |
| “Male nurses are very supportive” (Sundus and Younas, 2020) |  |  |
| “Protecting patients from physical harm ( fall , ulcers, malnutrition)”(Dobrowolska and Palese, 2016; Esmaeili et al., 2016; Andersson et al., 2015)  Patients should receive accurate and safe care and reduce medical errors (Fang et al., 2020). | Assurance |  |
| “Keep checking to make sure we’re ok” (Dobrowolska and Palese, 2016; Mako et al., 2016; Amy et al., 2012) |  |  |
| Being with/presence (Dobrowolska and Palese, 2016; Thomas et al., 2019) |  |  |
| “Making patients sure that they are not alone with their problems” (Dobrowolska and Palese, 2016) |  |  |
| “Ensure patients feel safe” (Dobrowolska and Palese, 2016; Cheruiyot and Brysiewicz, 2019; Mako et al., 2016; Tsai, Y.C. and Wang, Y.H., 2015) |  |  |
| “Reduce patients’ anxiety when facing invasive treatments”(Mako et al., 2016; Tsai, Y.C. and Wang, Y.H., 2015) |  |  |
| “Early identification of complications” (Petrou et al., 2017; Tsai, Y.-C. and Wang, Y.-H., 2015) |  |  |
| “Avoid placing the patient’s life in danger” (Tsai, Y.C. and Wang, Y.H., 2015)  Nurses should “be self-motivated and attentive to details and perform duties carefully.”(Fang et al., 2020) |  |  |
| “Sense of worth and identity only when nurses frequently visited them and evaluated their complaints”(Canzan et al., 2014) |  |  |
| A nurse is a competent, responsible and an authentic person. Accountable for their action and observer. A nurse need to be altruistic, committed, dedicated (Fang et al., 2020).  “I am willing to work tirelessly for the comfort of patients.”(Fang et al., 2020).  Nurses should take care of patients holistically (Fang et al., 2020). |  |  |
| Allow me to choose the best time to talk about my concerns (Thomas et al., 2019; Sundus and Younas, 2020) | Connectedness    Respect | Psychosocial Care |
| Frequently visiting the patient to inquire about needs (Sundus and Younas, 2020) |  |  |
| Spending extra time at the bedside (Sundus and Younas, 2020) |  |  |
| Collaborating with HCP to manage patient issues (Sundus and Younas, 2020)  “Nurses felt that in order to help patients and their families, nurses should help colleagues and the units as much as possible within their capabilities”(Fang et al., 2020).  “Advocate for patients and families and consider the most feasible care plans” (Fang et al., 2020) |  |  |
|  |  |  |
|  |  |  |
| Planning of the patient (Thomas et al., 2019) |  |  |
| Spending time with the patient (Amy et al., 2012; Dobrowolska and Palese, 2016; Pearcey, P., 2010) |  |  |
| Act of listening to people (Dobrowolska and Palese, 2016; Canzan et al., 2014; Esmaeili et al., 2016; Modic et al., 2014; Pearcey, P., 2010; Rahbel et al., 2019; Mako et al., 2016; Pearcey, Patricia, 2010; Thomas et al., 2019) “allow patients to express their feelings” (Fang et al., 2020) |  |  |
| Staff that were easy to talk (Pearcey, P., 2010; Mako et al., 2016) |  |  |
| Hold their hand, touch their arm and forehead (Pearcey, P., 2010; Petrou et al., 2017) |  |  |
| Offering emotional support and/or counselling (Petrou et al., 2017; Phillips et al., 2015; Sundus and Younas, 2020) |  |  |
| Nurses advocating on his/her behalf to the rest of the team (Canzan et al., 2014; Sundus and Younas, 2020) |  |  |
| Involvement of family in taking care of patients (Jardien-Baboo et al., 2016) |  |  |
| Kept a promise (Canzan et al., 2014) |  |  |
| Shared personal health experiences(Modic et al., 2014) |  |  |
| Nurses should be aware of their verbal and non-verbal communication(Jardien-Baboo et al., 2016; Costello, 2017; Canzan et al., 2014; Kalfoss, M. et al., 2017 ; Esmaeili et al., 2016; Modic et al., 2014; Rahbel et al., 2019; Fang et al., 2020) |  |  |
| Caring gesture: smile, tone should not be loud and respectful, polite (Sundus and Younas, 2020) |  |  |
| Apologising for late response to call for care (Sundus and Younas, 2020) |  |  |
| use of simple language (Canzan et al., 2014) |  |  |
| Use of humor while providing care to the patients (Amy et al., 2012) |  |  |
| Inspiring hope in patients (Cheruiyot and Brysiewicz, 2019; Thomas et al., 2019) |  |  |
| Help me deal with my bad feelings (Thomas et al., 2019) |  |  |
| Respecting their needs and encouraging self-respect (Kalfoss, Mary and Owe, 2017; Sundus and Younas, 2020) |  |  |
| Respecting emotions, feelings, preference (Sundus and Younas, 2020) |  |  |
| Respect my need for privacy (Sundus and Younas, 2020; Thomas et al., 2019) |  |  |
| Show respect for those things that have meaning to me (Thomas et al., 2019) |  |  |
| Respect for the uniqueness of the other(Canzan et al., 2014; Kalfoss, M. et al., 2017 ) |  |  |
| Empathetic to patients emotions (Sundus and Younas, 2020)  “Have love and sympathy towards patients and families. Embrace and respect patients’ human right and dignity”(Fang et al., 2020)  “Treat patients as family members” (Fang et al., 2020)  Compassionnate (Fang et al., 2020) |  |  |
| Need to be treated as an equal (Amy et al., 2012; Sundus and Younas, 2020) |  |  |
| Think before reacting to patients’ negative comments. Understand patient viewpoint (Sundus and Younas, 2020) |  |  |
| Making the patient feel like a human being (Dobrowolska and Palese, 2016) |  |  |
| Nurses who were ‘friendly’, ‘warm’, ‘kind’, ‘concerned’ and ‘sensitive (Canzan et al., 2014; Sundus and Younas, 2020) |  |  |
| Awareness of the patient's culture (Jardien-Baboo et al., 2016; Cheruiyot and Brysiewicz, 2019; Kalfoss, M. et al., 2017 ) |  |  |
| Right to make independent decision making (Amy et al., 2012; Dobrowolska and Palese, 2016; Canzan et al., 2014; Esmaeili et al., 2016; Mako et al., 2016) |  |  |
| Nurses should not be too stringent and inattentive to patients’ rational expectations (Esmaeili et al., 2016) |  |  |
| Enhance autonomy (Kalfoss, M. et al., 2017 ) |  |  |
| Autonomy was not respected (Sundus and Younas, 2020) |  |  |
| Fulfilling patient feasible requests (Dobrowolska and Palese, 2016) |  |  |
| Improving patient independency (Dobrowolska and Palese, 2016) |  |  |
| Caring for the spiritual needs of patients(Dobrowolska and Palese, 2016; Kalfoss, M. et al., 2017 ; Costello, 2017)  “Respect patients, understand their feelings and give spiritual encouragement when they feel helpless”(Fang et al., 2020) |  |  |
| Establishing trusting relationship, experience a sense of safety, worth, and identity (Esmaeili et al., 2016; Sundus and Younas, 2020; Fang et al., 2020) | Trusting Relationship |  |
| Providing information to the patient and family members(Canzan et al., 2014; Andersson et al., 2015; Esmaeili et al., 2016; Modic et al., 2014; Rahbel et al., 2019; Mako et al., 2016) | Teaching and Learning |  |
| Providing feedback(Thomas et al., 2019) |  |  |

**Table 2** Thematic analysis of qualitative data

| **Codes** | **Descriptive themes** | **Analytical Themes** |
| --- | --- | --- |
| Reflection and critical thinking (Canzan et al., 2014) | Knowledge and Skills | Physical Care |
| Thinking about patient’s needs (Canzan et al., 2014) addressing patient’s needs (Pearcey, P., 2010) |  |  |
| Evaluating the care being provided (Canzan et al., 2014) |  |  |
| “Strong knowledge about different conditions of patients with different diseases” (Canzan et al., 2014; Sundus and Younas, 2020) |  |  |
| Promptly recognised changes (signs and symptoms) (Canzan et al., 2014) |  |  |
| “Caring is about to find the peoples’ problem and solve it (Dobrowolska and Palese, 2016; Pearcey, P., 2010). |  |  |
| “Assessing vital signs and physiological changes”(Jill et al., 2015; Andersson et al., 2015) |  |  |
| “Understand the patient’s body language” (Andersson et al., 2015) |  |  |
| Updated knowledge and clinical skills (Kalfoss, M. et al., 2017 ; Andersson et al., 2015) |  |  |
| “Collecting data about the patients to form a basis for diagnosis, assessment and action” (Andersson et al., 2015) |  |  |
| “Pharmacological intervention, medication and non-pharmacological actions ‘touching’ and ‘changing the patient’s position’ to relieve symptoms” (Jill et al., 2015; Andersson et al., 2015). |  |  |
| Evaluating the effects of different types of treatment (Andersson et al., 2015) |  |  |
| Caring as the ‘extra’ or  ‘extra mile’(Dobrowolska and Palese, 2016; Kalfoss, M. et al., 2017 ; Amy et al., 2012; Pearcey, P., 2010) |  |  |
| “Providing physical comfort patient felt higher satisfaction” (Thomas et al., 2019; Tsai, Y.-C. and Wang, Y.-H., 2015) | Comfort |  |
| Helping to perform basic daily activities ( feeding, bathing, toileting) (Andersson et al., 2015; Canzan et al., 2014; Dobrowolska and Palese, 2016; Petrou et al., 2017; Thomas et al., 2019) |  |  |
| “Treat my body carefully” (Thomas et al., 2019) |  |  |
| “Ward and room environment comfortable” (Sundus and Younas, 2020) |  |  |
| “Administration of medication, changing patients’ position, offer things such as blanket, keep the things within reach of the patients, providing backrub, making the surrounding neat and clean before leaving the room” (Andersson et al., 2015; Canzan et al., 2014; Dobrowolska and Palese, 2016; Edvardsson et al., 2017; Omari et al., 2013; Phillips et al., 2015; Tsai, Y.-C. and Wang, Y.-H., 2015; Youssef et al., 2013) |  |  |
| Relieving physical pain (Petrou et al., 2017) |  |  |
| “Male nurses are very supportive” (Sundus and Younas, 2020) |  |  |
| “Protecting patients from physical harm ( fall , ulcers, malnutrition)”(Dobrowolska and Palese, 2016; Esmaeili et al., 2016; Andersson et al., 2015) | Assurance |  |
| “Keep checking to make sure we’re ok” (Dobrowolska and Palese, 2016; Mako et al., 2016; Amy et al., 2012) |  |  |
| Being with/presence (Dobrowolska and Palese, 2016; Thomas et al., 2019) |  |  |
| “Making patients sure that they are not alone with their problems” (Dobrowolska and Palese, 2016) |  |  |
| “Ensure patients feel safe” (Dobrowolska and Palese, 2016; Cheruiyot and Brysiewicz, 2019; Mako et al., 2016; Tsai, Y.C. and Wang, Y.H., 2015) |  |  |
| “Reduce patients’ anxiety when facing invasive treatments”(Mako et al., 2016; Tsai, Y.C. and Wang, Y.H., 2015) |  |  |
| “Early identification of complications” (Petrou et al., 2017; Tsai, Y.-C. and Wang, Y.-H., 2015) |  |  |
| “Avoid placing the patient’s life in danger” (Tsai, Y.C. and Wang, Y.H., 2015) |  |  |
| “Sense of worth and identity only when nurses frequently visited them and evaluated their complaints”(Canzan et al., 2014) |  |  |
| Allow me to choose the best time to talk about my concerns (Thomas et al., 2019; Sundus and Younas, 2020) | Connectedness    Respect | Psychosocial Care |
| Frequently visiting the patient to inquire about needs (Sundus and Younas, 2020) |  |  |
| Spending extra time at the bedside (Sundus and Younas, 2020) |  |  |
| Collaborating with HCP to manage patient issues (Sundus and Younas, 2020) |  |  |
|  |  |  |
|  |  |  |
| Planning of the patient (Thomas et al., 2019) |  |  |
| Spending time with the patient (Amy et al., 2012; Dobrowolska and Palese, 2016; Pearcey, P., 2010) |  |  |
| Act of listening to people (Dobrowolska and Palese, 2016; Canzan et al., 2014; Esmaeili et al., 2016; Modic et al., 2014; Pearcey, P., 2010; Rahbel et al., 2019; Mako et al., 2016; Pearcey, Patricia, 2010; Thomas et al., 2019; Fang et al., 2020) |  |  |
| Staff that were easy to talk (Pearcey, P., 2010; Mako et al., 2016) |  |  |
| Hold their hand, touch their arm and forehead (Pearcey, P., 2010; Petrou et al., 2017) |  |  |
| Offering emotional support and/or counselling (Petrou et al., 2017; Phillips et al., 2015; Sundus and Younas, 2020) |  |  |
| Nurses advocating on his/her behalf to the rest of the team (Canzan et al., 2014; Sundus and Younas, 2020) |  |  |
| Involvement of family in taking care of patients (Jardien-Baboo et al., 2016) |  |  |
| Kept a promise (Canzan et al., 2014) |  |  |
| Shared personal health experiences(Modic et al., 2014) |  |  |
| Nurses should be aware of their verbal and non-verbal communication(Jardien-Baboo et al., 2016; Costello, 2017; Canzan et al., 2014; Kalfoss, M. et al., 2017 ; Esmaeili et al., 2016; Modic et al., 2014; Rahbel et al., 2019) |  |  |
| Caring gesture: smile, tone should not be loud and respectful, polite (Sundus and Younas, 2020) |  |  |
| Apologising for late response to call for care (Sundus and Younas, 2020) |  |  |
| use of simple language (Canzan et al., 2014) |  |  |
| Use of humor while providing care to the patients (Amy et al., 2012) |  |  |
| Inspiring hope in patients (Cheruiyot and Brysiewicz, 2019; Thomas et al., 2019) |  |  |
| Help me deal with my bad feelings (Thomas et al., 2019) |  |  |
| Respecting their needs and encouraging self-respect (Kalfoss, Mary and Owe, 2017; Sundus and Younas, 2020) |  |  |
| Respecting emotions, feelings, preference (Sundus and Younas, 2020) |  |  |
| Respect my need for privacy (Sundus and Younas, 2020; Thomas et al., 2019) |  |  |
| Show respect for those things that have meaning to me (Thomas et al., 2019) |  |  |
| Respect for the uniqueness of the other(Canzan et al., 2014; Kalfoss, M. et al., 2017 ) |  |  |
| Empathetic to patients emotions (Sundus and Younas, 2020) |  |  |
| Need to be treated as an equal (Amy et al., 2012; Sundus and Younas, 2020) |  |  |
| Think before reacting to patients’ negative comments. Understand patient viewpoint (Sundus and Younas, 2020) |  |  |
| Making the patient feel like a human being (Dobrowolska and Palese, 2016) |  |  |
| Nurses who were ‘friendly’, ‘warm’, ‘kind’, ‘concerned’ and ‘sensitive (Canzan et al., 2014; Sundus and Younas, 2020) |  |  |
| Awareness of the patient's culture (Jardien-Baboo et al., 2016; Cheruiyot and Brysiewicz, 2019; Kalfoss, M. et al., 2017 ) |  |  |
| Right to make independent decision making (Amy et al., 2012; Dobrowolska and Palese, 2016; Canzan et al., 2014; Esmaeili et al., 2016; Mako et al., 2016) |  |  |
| Nurses should not be too stringent and inattentive to patients’ rational expectations (Esmaeili et al., 2016) |  |  |
| Enhance autonomy (Kalfoss, M. et al., 2017 ) |  |  |
| Autonomy was not respected (Sundus and Younas, 2020) |  |  |
| Fulfilling patient feasible requests (Dobrowolska and Palese, 2016) |  |  |
| Improving patient independency (Dobrowolska and Palese, 2016) |  |  |
| Caring for the spiritual needs of patients(Dobrowolska and Palese, 2016; Kalfoss, M. et al., 2017 ; Costello, 2017) |  |  |
| Establishing trusting relationship, experience a sense of safety, worth, and identity (Esmaeili et al., 2016; Sundus and Younas, 2020) | Trusting Relationship |  |
| Providing information to the patient and family members(Canzan et al., 2014; Andersson et al., 2015; Esmaeili et al., 2016; Modic et al., 2014; Rahbel et al., 2019; Mako et al., 2016) | Teaching and Learning |  |
| Providing feedback(Thomas et al., 2019) |  |  |

**Table 2** Thematic analysis of qualitative data

| **Codes** | **Descriptive themes** | **Analytical Themes** |
| --- | --- | --- |
| Reflection and critical thinking (Canzan et al., 2014) | Knowledge and Skills | Physical Care |
| Thinking about patient’s needs (Canzan et al., 2014) addressing patient’s needs (Pearcey, P., 2010) |  |  |
| Evaluating the care being provided (Canzan et al., 2014) |  |  |
| “Strong knowledge about different conditions of patients with different diseases” (Canzan et al., 2014; Sundus and Younas, 2020) |  |  |
| Promptly recognised changes (signs and symptoms) (Canzan et al., 2014) |  |  |
| “Caring is about to find the peoples’ problem and solve it (Dobrowolska and Palese, 2016; Pearcey, P., 2010). |  |  |
| “Assessing vital signs and physiological changes”(Jill et al., 2015; Andersson et al., 2015) |  |  |
| “Understand the patient’s body language” (Andersson et al., 2015) |  |  |
| Updated knowledge and clinical skills (Kalfoss, M. et al., 2017 ; Andersson et al., 2015) |  |  |
| “Collecting data about the patients to form a basis for diagnosis, assessment and action” (Andersson et al., 2015) |  |  |
| “Pharmacological intervention, medication and non-pharmacological actions ‘touching’ and ‘changing the patient’s position’ to relieve symptoms” (Jill et al., 2015; Andersson et al., 2015). |  |  |
| Evaluating the effects of different types of treatment (Andersson et al., 2015) |  |  |
| Caring as the ‘extra’ or  ‘extra mile’(Dobrowolska and Palese, 2016; Kalfoss, M. et al., 2017 ; Amy et al., 2012; Pearcey, P., 2010) |  |  |
| “Providing physical comfort patient felt higher satisfaction” (Thomas et al., 2019; Tsai, Y.-C. and Wang, Y.-H., 2015) | Comfort |  |
| Helping to perform basic daily activities ( feeding, bathing, toileting) (Andersson et al., 2015; Canzan et al., 2014; Dobrowolska and Palese, 2016; Petrou et al., 2017; Thomas et al., 2019) |  |  |
| “Treat my body carefully” (Thomas et al., 2019) |  |  |
| “Ward and room environment comfortable” (Sundus and Younas, 2020) |  |  |
| “Administration of medication, changing patients’ position, offer things such as blanket, keep the things within reach of the patients, providing backrub, making the surrounding neat and clean before leaving the room” (Andersson et al., 2015; Canzan et al., 2014; Dobrowolska and Palese, 2016; Edvardsson et al., 2017; Omari et al., 2013; Phillips et al., 2015; Tsai, Y.-C. and Wang, Y.-H., 2015; Youssef et al., 2013) |  |  |
| Relieving physical pain (Petrou et al., 2017) |  |  |
| “Male nurses are very supportive” (Sundus and Younas, 2020) |  |  |
| “Protecting patients from physical harm ( fall , ulcers, malnutrition)”(Dobrowolska and Palese, 2016; Esmaeili et al., 2016; Andersson et al., 2015) | Assurance |  |
| “Keep checking to make sure we’re ok” (Dobrowolska and Palese, 2016; Mako et al., 2016; Amy et al., 2012) |  |  |
| Being with/presence (Dobrowolska and Palese, 2016; Thomas et al., 2019) |  |  |
| “Making patients sure that they are not alone with their problems” (Dobrowolska and Palese, 2016) |  |  |
| “Ensure patients feel safe” (Dobrowolska and Palese, 2016; Cheruiyot and Brysiewicz, 2019; Mako et al., 2016; Tsai, Y.C. and Wang, Y.H., 2015) |  |  |
| “Reduce patients’ anxiety when facing invasive treatments”(Mako et al., 2016; Tsai, Y.C. and Wang, Y.H., 2015) |  |  |
| “Early identification of complications” (Petrou et al., 2017; Tsai, Y.-C. and Wang, Y.-H., 2015) |  |  |
| “Avoid placing the patient’s life in danger” (Tsai, Y.C. and Wang, Y.H., 2015) |  |  |
| “Sense of worth and identity only when nurses frequently visited them and evaluated their complaints”(Canzan et al., 2014) |  |  |
| Allow me to choose the best time to talk about my concerns (Thomas et al., 2019; Sundus and Younas, 2020) | Connectedness    Respect | Psychosocial Care |
| Frequently visiting the patient to inquire about needs (Sundus and Younas, 2020) |  |  |
| Spending extra time at the bedside (Sundus and Younas, 2020) |  |  |
| Collaborating with HCP to manage patient issues (Sundus and Younas, 2020) |  |  |
|  |  |  |
|  |  |  |
| Planning of the patient (Thomas et al., 2019) |  |  |
| Spending time with the patient (Amy et al., 2012; Dobrowolska and Palese, 2016; Pearcey, P., 2010) |  |  |
| Act of listening to people (Dobrowolska and Palese, 2016; Canzan et al., 2014; Esmaeili et al., 2016; Modic et al., 2014; Pearcey, P., 2010; Rahbel et al., 2019; Mako et al., 2016; Pearcey, Patricia, 2010; Thomas et al., 2019) |  |  |
| Staff that were easy to talk (Pearcey, P., 2010; Mako et al., 2016) |  |  |
| Hold their hand, touch their arm and forehead (Pearcey, P., 2010; Petrou et al., 2017) |  |  |
| Offering emotional support and/or counselling (Petrou et al., 2017; Phillips et al., 2015; Sundus and Younas, 2020) |  |  |
| Nurses advocating on his/her behalf to the rest of the team (Canzan et al., 2014; Sundus and Younas, 2020) |  |  |
| Involvement of family in taking care of patients (Jardien-Baboo et al., 2016) |  |  |
| Kept a promise (Canzan et al., 2014) |  |  |
| Shared personal health experiences(Modic et al., 2014) |  |  |
| Nurses should be aware of their verbal and non-verbal communication(Jardien-Baboo et al., 2016; Costello, 2017; Canzan et al., 2014; Kalfoss, M. et al., 2017 ; Esmaeili et al., 2016; Modic et al., 2014; Rahbel et al., 2019) |  |  |
| Caring gesture: smile, tone should not be loud and respectful, polite (Sundus and Younas, 2020) |  |  |
| Apologising for late response to call for care (Sundus and Younas, 2020) |  |  |
| use of simple language (Canzan et al., 2014) |  |  |
| Use of humor while providing care to the patients (Amy et al., 2012) |  |  |
| Inspiring hope in patients (Cheruiyot and Brysiewicz, 2019; Thomas et al., 2019) |  |  |
| Help me deal with my bad feelings (Thomas et al., 2019) |  |  |
| Respecting their needs and encouraging self-respect (Kalfoss, Mary and Owe, 2017; Sundus and Younas, 2020) |  |  |
| Respecting emotions, feelings, preference (Sundus and Younas, 2020) |  |  |
| Respect my need for privacy (Sundus and Younas, 2020; Thomas et al., 2019) |  |  |
| Show respect for those things that have meaning to me (Thomas et al., 2019) |  |  |
| Respect for the uniqueness of the other(Canzan et al., 2014; Kalfoss, M. et al., 2017 ) |  |  |
| Empathetic to patients emotions (Sundus and Younas, 2020) |  |  |
| Need to be treated as an equal (Amy et al., 2012; Sundus and Younas, 2020) |  |  |
| Think before reacting to patients’ negative comments. Understand patient viewpoint (Sundus and Younas, 2020) |  |  |
| Making the patient feel like a human being (Dobrowolska and Palese, 2016) |  |  |
| Nurses who were ‘friendly’, ‘warm’, ‘kind’, ‘concerned’ and ‘sensitive (Canzan et al., 2014; Sundus and Younas, 2020) |  |  |
| Awareness of the patient's culture (Jardien-Baboo et al., 2016; Cheruiyot and Brysiewicz, 2019; Kalfoss, M. et al., 2017 ) |  |  |
| Right to make independent decision making (Amy et al., 2012; Dobrowolska and Palese, 2016; Canzan et al., 2014; Esmaeili et al., 2016; Mako et al., 2016) |  |  |
| Nurses should not be too stringent and inattentive to patients’ rational expectations (Esmaeili et al., 2016) |  |  |
| Enhance autonomy (Kalfoss, M. et al., 2017 ) |  |  |
| Autonomy was not respected (Sundus and Younas, 2020) |  |  |
| Fulfilling patient feasible requests (Dobrowolska and Palese, 2016) |  |  |
| Improving patient independency (Dobrowolska and Palese, 2016) |  |  |
| Caring for the spiritual needs of patients(Dobrowolska and Palese, 2016; Kalfoss, M. et al., 2017 ; Costello, 2017) |  |  |
| Establishing trusting relationship, experience a sense of safety, worth, and identity (Esmaeili et al., 2016; Sundus and Younas, 2020) | Trusting Relationship |  |
| Providing information to the patient and family members(Canzan et al., 2014; Andersson et al., 2015; Esmaeili et al., 2016; Modic et al., 2014; Rahbel et al., 2019; Mako et al., 2016) | Teaching and Learning |  |
| Providing feedback(Thomas et al., 2019) |  |  |

Amy, M., Alison, K. and Kathryn, Z. 2012. Patients’ views of patient‐centred care: a phenomenological case study in one surgical unit. *Journal of Advanced Nursing.* **68**(12), pp.2664-2673.

Andersson, E.K., Willman, A., Sjöström-Strand, A. and Borglin, G. 2015. Registered nurses’ descriptions of caring: a phenomenographic interview study. *BMC nursing.* **14**(1), p16.

Canzan, F., Heilemann, M.V., Saiani, L., Mortari, L. and Ambrosi, E. 2014. Visible and invisible caring in nursing from the perspectives of patients and nurses in the gerontological context. *Scandinavian journal of caring sciences.* **28**(4), pp.732-740.

Cheruiyot, J.C. and Brysiewicz, P. 2019. Nurses’ perceptions of caring and uncaring nursing encounters in inpatient rehabilitation settings in South Africa: A qualitative descriptive study. *International Journal of Africa Nursing Sciences.* **11**, p100160.

Costello, M. 2017. Nurses’ Self-Identified Characteristics and Behaviors Contributing to Patients’ Positive Perceptions of Their Nursing Care: A Qualitative Study. *Journal of Holistic Nursing.* **35**(1), pp.62-66.

Dobrowolska, B. and Palese, A. 2016. The caring concept, its behaviours and obstacles: perceptions from a qualitative study of undergraduate nursing students. *Nursing inquiry.* **23**(4), pp.305-314.

Edvardsson, D., Watt, E. and Pearce, F. 2017. Patient experiences of caring and person‐centredness are associated with perceived nursing care quality. *Journal of Advanced Nursing.* **73**(1), pp.217-227.

Esmaeili, M., Cheraghi, M.A. and Salsali, M. 2016. Cardiac patients' perception of patient‐centred care: a qualitative study. *Nursing in critical care.* **21**(2), pp.97-104.

Fang, F., Zhu, H., Li, X. and Wei, H. 2020. Nurses' perceptions of caring: a directed content analysis based on the care model. *International Journal for Human Caring.* **24**(1), pp.50-58.

Jardien-Baboo, S., van Rooyen, D., Ricks, E. and Jordan, P. 2016. Perceptions of patient-centred care at public hospitals in Nelson Mandela Bay. *health sa gesondheid.* **21**(1), pp.397-405.

Jill, P., Karen, C., Elizabeth, R., Janet, S., Vanessa, H., Sara, W., Ian, D., Eleanor, J., Ann, H. and Andrew, H. 2015. An exploration of the perceptions of caring held by students entering nursing programmes in the United Kingdom: A longitudinal qualitative study phase 1. *Nurse education in practice.* **15**(6), pp.403-408.

Kalfoss, M. and Owe, J. 2017. Meanings given to professional care: focus group results.

Kalfoss, M., Owe, J. and Scient, C. 2017 Meanings Given to Professional Care :Focus Group Results

*Open Journal of Nursing.* **7**, pp.524-547.

Mako, T., Svanäng, P. and Bjerså, K. 2016. Patients’ perceptions of the meaning of good care in surgical care: a grounded theory study. *BMC nursing.* **15**(1), p47.

Modic, M.B., Siedlecki, S.L., Griffin, M.T.Q. and Fitzpatrick, J.J. 2014. Caring behaviors: Perceptions of acute care

nurses and hospitalized patients with diabetes

*Journal of Patient Experience.* **1**(1), pp.26-30.

Omari, F.H., AbuAlRub, R. and Ayasreh, I.R. 2013. Perceptions of patients and nurses towards nurse caring behaviors in coronary care units in J ordan. *Journal of clinical nursing.* **22**(21-22), pp.3183-3191.

Pearcey, P. 2010. Caring? It's the little things we are not supposed to do anymore. *International Journal of Nursing Practice.* **16**(1), pp.51-56.

Pearcey, P. 2010. Caring? It's the little things we are not supposed to do anymore. *International Journal of Nursing Practice.* **16**, pp.51– 56.

Petrou, A., Sakellari, E., Psychogiou, M., Karassavidis, S., Imbrahim, S., Savvidis, G. and Sapountzi-Krepia, D. 2017. Nursing students’ perceptions of caring: A qualitative approach. *International Journal of Caring Sciences.* **10**(3), pp.1148-1157.

Phillips, J., Cooper, K., Rosser, E., Scammell, J., Heaslip, V., White, S., Donaldson, I., Jack, E., Hemingway, A. and Harding, A. 2015. An exploration of the perceptions of caring held by students entering nursing programmes in the United Kingdom: A longitudinal qualitative study phase 1. *Nurse education in practice.* **15**(6), pp.403-408.

Rahbel, R., Elizabeth, B.M., Alizeh, A., Mahnoor, R.S., Umme, S., Lubna, S. and Mansoor, K. 2019. Perceptions of patient‐centred care among providers and patients in the orthopaedic department of a tertiary care hospital in Karachi, Pakistan. *Journal of evaluation in clinical practice.* **25**(6), pp.1160-1168.

Sundus, A. and Younas, A. 2020. Caring behaviors of male nurses: A descriptive qualitative study of patients' perspectives. In: *Nursing Forum*: Wiley Online Library, pp.575-581.

Thomas, D., Newcomb, P. and Fusco, P. 2019. Perception of caring among patients and nurses. *Journal of patient experience.* **6**(3), pp.194-200.

Tsai, Y.-C. and Wang, Y.-H. 2015. Caring behavior exhibited by Taiwanese nurses. *International Journal of Caring Sciences.* **8**(2), p317.

Tsai, Y.C. and Wang, Y.H. 2015. Caring behavior exhibited by Taiwanese nurses. *International Journal of Caring Sciences.* **8**(2), p317.

Youssef, H.A., Mansour, M.A., Ayasreh, I.R. and Al-Mawajdeh, N.A. 2013. A Medical-Surgical Nurse's Perceptions of Caring Behaviors among Hospitals in Taif City. *Life Science Journal.* **10**(4), pp.720-730.
